# Temperature-related mortality in China from specific injury

**DOI:** 10.1038/s41467-022-35462-4

**Published:** 2023-01-03

**Authors:** Jianxiong Hu, Guanhao He, Ruilin Meng, Weiwei Gong, Zhoupeng Ren, Heng Shi, Ziqiang Lin, Tao Liu, Fangfang Zeng, Peng Yin, Guoxia Bai, Mingfang Qin, Zhulin Hou, Xiaomei Dong, Chunliang Zhou, Zhuoma Pingcuo, Yize Xiao, Min Yu, Biao Huang, Xiaojun Xu, Lifeng Lin, Jianpeng Xiao, Jieming Zhong, Donghui Jin, Qinglong Zhao, Yajie Li, Cangjue Gama, Yiqing Xu, Lingshuang Lv, Weilin Zeng, Xing Li, Liying Luo, Maigeng Zhou, Cunrui Huang, Wenjun Ma

**Affiliations:** 1 Guangdong Provincial Institute of Public Health, Guangdong Provincial Center for Disease Control and Prevention, Guangzhou, 511430 China; 2 Department of Public Health and Preventive Medicine, School of Medicine, Jinan University, Guangzhou, 511443 China; 3 Guangdong Provincial Center for Disease Control and Prevention, Guangzhou, 511430 China; 4 Zhejiang Provincial Center for Disease Control and Prevention, Hangzhou, 310009 China; 5 State Key Laboratory of Resources and Environmental Information System, Institute of Geographic Sciences and Natural Resources Research, Chinese Academy of Sciences, Beijing, 100101 China; 6 Tibet Autonomous Region Center for Disease Control and Prevention, Lhasa, 850002 China; 7 The National Center for Chronic and Noncommunicable Disease Control and Prevention, Beijing, 100050 China; 8 Yunnan Provincial Center for Disease Control and Prevention, Kunming, 650034 China; 9 Jilin Provincial Center for Disease Control and Prevention, Changchun, 130062 China; 10 Hunan Provincial Center for Disease Control and Prevention, Changsha, 410005 China; 11 Vanke School of Public Health, Tsinghua University, Beijing, 100084 China

**Keywords:** Risk factors, Environmental health

## Abstract

Injury poses heavy burden on public health, accounting for nearly 8% of all deaths globally, but little evidence on the role of climate change on injury exists. We collect data during 2013-2019 in six provinces of China to examine the effects of temperature on injury mortality, and to project future mortality burden attributable to temperature change driven by climate change based on the assumption of constant injury mortality and population scenario. The results show that a 0.50% (95% confident interval (CI): 0.13%–0.88%) increase of injury mortality risk for each 1 °C rise in daily temperature, with higher risk for intentional injury (1.13%, 0.55%–1.71%) than that for unintentional injury (0.40%, 0.04%–0.77%). Compared to the 2010s, total injury deaths attributable to temperature change in China would increase 156,586 (37,654–272,316) in the 2090 s under representative concentration pathways 8.5 scenario with the highest for transport injury (64,764, 8,517–115,743). Populations living in Western China, people aged 15–69 years, and male may suffer more injury mortality burden from increased temperature caused by climate change. Our findings may be informative for public health policy development to effectively adapt to climate change.

## Abstract (web summary)

Injury poses heavy burden on public health, but little evidence on the potential role of climate change on injury exists. Here, the authors collect data during 2013-2019 in six provinces of China to estimate the associations between temperature and injury mortality, and to project future mortality burden attributable to temperature change driven by climate change.

## Introduction

Injury is a crucial public health challenge that affects every country in the world. The World Health Organization estimated that 4.4 million people worldwide die from injury each year, accounting for nearly 8.0% of total deaths^1^. Injury is also seen as a serious public health concern in China. With rapid economic development, China has been experiencing a decrease in mortality rates and disability adjusted life years (DALYs) due to injury in the past three decades. However, injury is still the fifth leading cause of death, accounting for 7.0% of total deaths and 10.0% of all-cause DALYs in China in 2017^2,3^.

A large number of factors and the complex interactions between these factors may contribute to injury, such as the use of alcohol or drug, inadequate adult supervision of children, lack of legislation, poverty, unemployment, etc.^1,4^. With climate change, the impact of climate on health is of great concern^5^, and the potential linkages between temperature and injury are increasingly being investigated^6–9^. The global burden of disease estimated that heat-related injury deaths accounted for 22% of heat-related all-cause deaths globally in 2019^10^, which has been downplayed by the governments and media through ignoring or misreporting the catastrophic health risk of extremely high temperature^11^. However, compared to non-injury causes such as non-communicable diseases and vector-borne infectious diseases, few researches have focused on the associations between climate and injury, especially in developing countries, where people simultaneously suffer heavy burden from both injury and climate change^12–15^. Moreover, existing studies mainly focused on single injury such as suicide, traffic injury, or fall, with fewer studies involving in mechanism-specific injuries and demographic subgroups^8,9^, and the full picture of the effects of temperature on injury remains unclear. Therefore, it is very necessary to conduct a comprehensive study to investigate the associations between ambient temperature and injury mortality in China, where people suffer from greater climate change and injury burden than the global levels^10,16^, and further assess the temperature-related injury mortality burden driven by climate change, because even small changes in injury mortality risk due to climate change can lead to large changes in the associated injury mortality burden.

In the present study, we used over 600,000 injury deaths from six provinces in China (Supplementary Fig. 1) to assess the association between temperature and injury mortality, and further to project the temperature-related injury mortality burden driven by climate change in the future. Our findings provided a comprehensive understanding of the relationship between temperature and injury nowadays and in the future, which may be informative for the governmental multi-sectors to work together and develop specific adaptation strategies targeted at injury, such as early warning system, environment modification and behavior change.

## Results and discussions

### Basic characteristics of the study sample

During the study period, there are 609,827 injury deaths in six provinces of China with 504,040(82.65%) unintentional cases and 75,893(12.45%) intentional cases. The number of injury deaths was much higher for males (403,701, 66.20%) than for females (206,104, 33.79%), and population aged over 50 years accounts for 59.50% of total injury deaths. The sex and age distributions of mechanism-specific injury were shown in Supplementary Table 1. For unintentional injury, traffic injury (31.55%), fall (25.56%), and drowning (7.59%) are the top three causes of injury death, while for intentional injury, suicide is the major injury (11.39%) (Table 1). The levels of environmental factors such as temperature, relative humidity and air pollutants between the case and control period were different for most mechanism-specific injuries (Supplementary Table 2), which indicates that these factors might be potential risk factors of injury.

**Table 1:** The basic characteristics of injury deaths in six provinces, China

Characteristics	Number	Proportion (%)
**Total injury deaths**	609827	100.0
**Sex**		
Male	403701	66.20
Female	206104	33.79
Unknow	22	0.01
**Age (year)**		
0–4	17905	2.94
5-14	16591	2.72
15-49	212483	34.84
50-69	167514	27.47
70+	195334	32.03
**Injury intention**		
**Unintentional injury**	504040	82.65
Transport injury	192424	31.55
Fall	155848	25.56
Drowning	46301	7.59
Poisoning	37671	6.18
Suffocation	21036	3.45
Mechanical force	28600	4.69
Other unintentional injury	22160	3.63
**Intentional injury**	75893	12.45
Suicide	69433	11.39
Assault	6460	1.06
**Other**	29894	4.90

### Association between ambient temperature and injury death

**Fig. 1:**
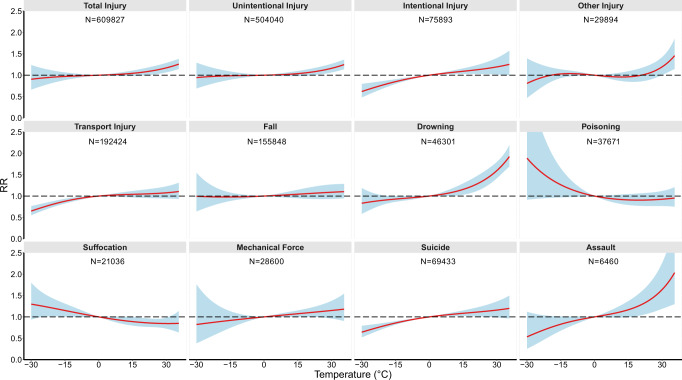
The exposure-response curves between daily mean temperature (over 0–1 lag days) and injury deaths by intention and mechanism. The solid lines (red) denote the relative risk of injury deaths compared to 0 °C. The shaded areas denote the 95% confidence interval. A conditional logistic regression with cross-basis function was used to estimate the province-specific temperature-injury deaths association adjusted for relative humidity, PM_2.5_ concentration and public holiday, which were pooled in a multivariate meta-analysis. The statistical tests were two-sided. RR, relative risk.

There was an approximately linear association between total injury or mechanism-specific injury mortality and daily mean temperature (Fig. 1). After linearization, we observed a 0.50% (95% CI: 0.13%–0.88%) increase for injury mortality for each 1 °C rise of daily mean temperature. Although some previous studies have reported that the association between temperature and injury was nonlinear^7,17^, there are many studies consistent with our findings^18–20^. For instance, a study from the global burden of disease reported that there was a positive linear relationship between ambient temperature and injury death in different temperature zone around the world^18^.

**Fig. 2:**
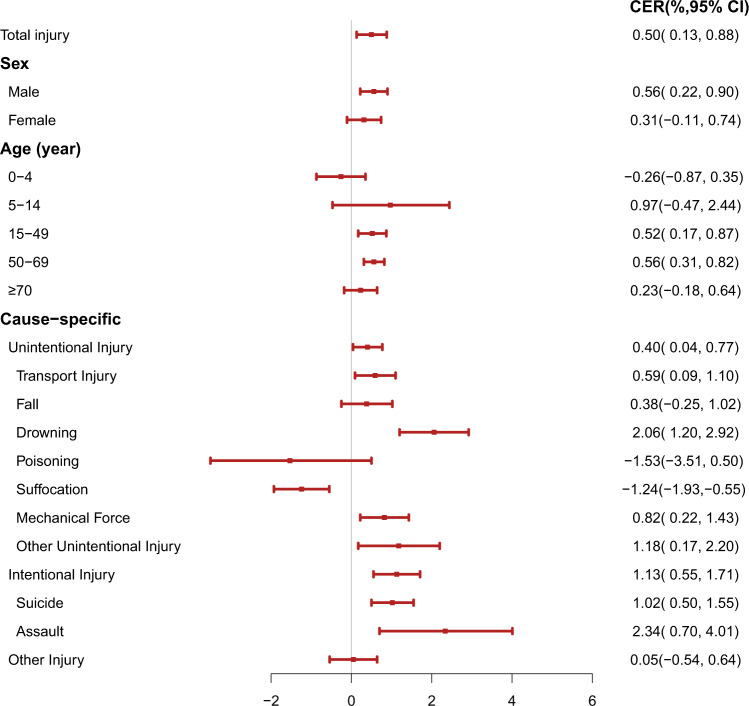
Cumulative excess risk(%) of injury deaths for each 1 °C increase in daily mean temperature over 0–1 lag days, by age, sex, intention and mechanism. The square red dots denote the cumulative excess risk (%). The horizontal red lines denote the 95% confidence interval. The light gray vertical line denotes the reference value (CER = 0). A conditional logistic regression with cross-basis function was used to estimate the province-specific temperature-injury deaths association adjusted for relative humidity, PM_2.5_ concentration and public holiday, which were pooled in a multivariate meta-analysis. The statistical tests were two-sided. CER, cumulative excess risk. *N* = 609827 independent samples of injury deaths were used to conduct 20 independent models; the exact sample sizes are shown as Supplementary Table 1.

For per 1 °C increase in daily mean temperature, the cumulative excess risk (CER) of unintentional injury increased by 0.40% (95% CI: 0.04%–0.77%). Specifically, drowning (CER = 2.06%, 95% CI: 1.20%–2.92%) had much higher mortality risk than transport injury (CER = 0.59%, 95% CI:0.09%–1.10%) and mechanic force (CER = 0.82%, 95% CI: 0.22%–1.43%) (Fig. 2). The underlying pathways for this phenomenon are complex and unclear. First, high temperatures, especially beyond the body’s ability to regulate, may reduce the performance on physical and intellectual tasks^21–23^. An experimental study showed that 5-hydroxytryptamine (5-HT), a marker of reduced decision-making capacity, was more prone to dysfunction in hot weather^24^, which may therefore increase the risk of unintentional injury events, such as traffic accidents, occupational injuries, and falls^25,26^. Second, some behaviors arise from high temperature may cause injury. Studies have shown that alcohol consumption increased on days with higher temperatures^27,28^, which may be an important factor of increased traffic injury. In addition, higher temperatures provide more opportunities for aquatic exposure, and people spend more time around the water, leading increased drowning^29,30^. Third, the performance of the equipment is inhibited by high temperatures, such as lower driving performance, larger steering adjustments of the vehicle, and loss of signals^25,31,32^, which might trigger traffic accidents or work injury. However, we also found a negative relationship between temperature and the mortality risk of accidental suffocation (CER = −1.24%,95% CI: −1.93%–−0.55%) and poisoning (CER = −1.53%,95% CI: −3.51%–0.50%). One possible reason might be that cold weather encourages people to live in sealed environment and take some measures to keep warm, such as using heating equipment and drinking spirits. These behaviors might increase the risk of insufficient oxygen or accidental poisoning^33,34^.

The CER of intentional injury was observed a 1.13% (95% CI: 0.55%–1.71%) increases per 1 °C increment in daily mean temperature, and assault (CER = 2.34%, 95% CI: 0.70%–4.01%) had much higher risk than suicide (CER = 1.02%, 95% CI: 0.50%–1.55%) (Fig. 2). This finding was similar to previous studies^35,36^. A case-crossover study found that every 5 °C increase in daily mean temperature will increase the risk of intentional homicide by 4.20% in the United States^35^. However, the pathway linking ambient temperature and intentional injury is different from unintentional injury. On the one hand, the effect of temperature on intentional injury may be explained by the biological theory, that is, high temperatures induce interpersonal violence or self-harm by increasing the emotions of discomfort, frustration, and impulsive aggression^37,38^. On the other hand, routine activity theory suggests that higher ambient temperatures may cause people to spend more time outdoor, thereby increasing the opportunities for face-to-face social contact and creating the environments for arguments, confrontation, or crime^39,40^.

A stratified analysis of mechanism-specific injury by sex and age found that there was large difference in temperature-related injury mortality risk among various age groups or sex. For example, children aged 0–4 years had greater risk of suffocation-related mortality (−3.10%, 95% CI: −4.39%–−1.81%), and populations aged 15–49 years had much higher risk of drowning mortality (3.41%, 95% CI: 2.27%–4.56%) than other age groups. Male had greater risk of temperature-related drowning, transport injury, and suicide mortality than female, while risk of assault and mechanic force mortality attributed to temperature was greater for female (Supplementary Fig. 2). The higher temperature-related suffocation mortality in children might be related to more warmth measures (e.g. more blankets and clothing) in winter, as accidental suffocation in bed is the leading cause of suffocation deaths in infants^41^. Young adults and male are more likely to expose themselves to dangerous water bodies at high temperature^42,43^. High temperature could also elevate the level of sex hormones^44^ and increase the risk of sexual offense^35^, which may lead to female being at higher risk of assault. However, these explanations of differential effects of temperature on specific injuries by age and sex remain preliminary and non-systematic, and more in-depth causal investigations should be conducted in the future. Our findings may be informative for the development of temperature-related injury risk early warning system. For instance, cold weather help warn suffocation and poisoning, while hot weather indicates higher drowning and traffic injury.

### Future injury mortality burden attributed to temperature change

Overall, compared to the 2010s, the temperature is projected to rise by 1.95 °C in the 2060 s, and 2.15 °C in the 2090 s under RCP4.5 scenario, while for RCP8.5 scenario, it is projected to rise by 2.90 °C and 4.98 °C, respectively (Supplementary Fig. 3). In terms of regional distribution, the highest increased temperatures were observed in western and northern China, with top three provinces in the 2090 s under the RCP8.5 scenario being Heilongjiang Province (5.91 °C), Tibet Autonomous Region (5.70 °C) and Xinjiang Uygur Autonomous Region (5.69 °C), respectively (Supplementary Fig. 4).

Compared with the 2010s, the total injury death caused by temperature change in China will increase from 61,348 (4.41/100000) in the 2060 s to 67,895 (4.88/100000) in the 2090 s under RCP4.5 scenario, while under RCP8.5 scenario, it will rise from 91,480 (6.58/100 000) in the 2060 s to 156,586 (11.26/100 000) in the 2090 s. These findings are consistent with a previous US national study, which found that excess injury deaths would increase by 2135 under a scenario of per 2 °C increase in average month temperature^45^.

The mortality burden of unintentional injury caused by temperature change were much greater than intentional injury during each period and climate change scenario. For example, from the 2060 s to the 2090 s under RCP8.5 scenario, the unintentional injury mortality rate caused by temperature change is from 4.37 (95% CI: 0.63–8.09) per 100,000 to 7.48 (95% CI: 1.08–13.83) per 100,000, while intentional injury mortality increased from 2.59 (95% CI: 1.22–3.92) to 4.41 (95% CI: 2.09–6.68) per 100,000. As for each injury, most mechanism-specific injuries will increase from the 2060 s to the 2090 s under both RCP4.5 and RCP8.5 scenarios, except for poisoning and suffocation. The top three projected injury mortality rates caused by temperature change are drowning, transport injury, and suicide, which are 4.83 (95% CI: 3.05–6.75), 4.66 (95% CI: 0.61–8.33), and 3.53 (95% CI: 1.77–5.25) per 100,000 in the 2090 s under RCP8.5 scenario (Table 2). Our findings suggest that future temperature increases may exacerbate most injury-related mortality burden, and that climate change mitigation and adaptation strategies are essential for injury prevention and control. Furthermore, in the context of irreversible climate change, comprehensive monitoring of temperature-related injury mortality burden should be enhanced to provide targeted early warning signals of health risks. In addition, strengthened the governments and media communication on health risk of climate change may be beneficial in improving individual health risk perception and adaptation skill.

**Table 2:** The projected mortality rate (per 100,000) of injury attributable to temperature change in the 2060 s and 2090 s compared to the 2010s under different RCP scenarios in China

	RCP4.5		RCP8.5	
	2060 s	2090 s	2060 s	2090 s
**Total Injury**	4.41(1.06,7.68)	4.88(1.17,8.51)	6.58(1.58,11.46)	11.26(2.71,19.59)
Male	6.91(2.96,11.23)	7.64(3.27,12.43)	10.3(4.41,16.75)	17.63(7.55,28.62)
Female	1.75(−0.61,4.12)	1.94(−0.67,4.56)	2.61(−0.9,6.14)	4.48(−1.55,10.50)
Age 0–4(y)	−1.58(−5.58,1.87)	−1.75(−6.16,2.07)	−2.35(−8.3,2.79)	−4.04(−14.32,4.78)
Age 5–14(y)	2.76(−1.24,6.52)	3.05(−1.37,7.24)	4.11(−1.85,9.75)	7.02(−3.18,16.53)
Age 15–49(y)	3.64(1.07,5.97)	4.02(1.18,6.61)	5.42(1.60,8.90)	9.28(2.74,15.22)
Age 50–69(y)	6.44(3.33,9.52)	7.13(3.68,10.55)	9.6(4.96,14.21)	16.43(8.49,24.29)
Age ≥ 70(y)	8.80(−6.21,24.42)	9.74(−6.87,27.03)	13.12(−9.25,36.42)	22.49(−15.89,62.32)
**Unintentional Injury**	2.93(0.42,5.42)	3.24(0.47,6.00)	4.37(0.63,8.09)	7.48(1.08,13.83)
Male	4.95(1.46,8.35)	5.48(1.62,9.24)	7.39(2.18,12.45)	12.64(3.74,21.28)
Female	0.75(−1.11,2.61)	0.83(−1.23,2.89)	1.12(−1.65,3.90)	1.92(−2.84,6.67)
Age 0–4(y)	−2.19(−5.79,1.56)	−2.42(−6.39,1.73)	−3.26(−8.61,2.33)	−5.60(−14.86,4.00)
Age 5–14(y)	2.34(−1.70,6.31)	2.59(−1.88,7.01)	3.49(−2.54,9.44)	5.96(−4.37,16.01)
Age 15–49(y)	2.23(0.13,4.34)	2.47(0.14,4.81)	3.32(0.19,6.48)	5.69(0.33,11.07)
Age 50–69(y)	4.59(2.58,6.66)	5.08(2.86,7.37)	6.84(3.85,9.93)	11.71(6.60,16.98)
Age ≥ 70(y)	3.99(−8.39,16.19)	4.42(−9.27,17.92)	5.95(−12.5,24.14)	10.21(−21.47,41.33)
Transport Injury	1.83(0.24,3.27)	2.02(0.27,3.62)	2.72(0.36,4.88)	4.66(0.61,8.33)
Fall	0.64(−0.37,1.72)	0.7(−0.41,1.91)	0.95(−0.55,2.57)	1.62(−0.95,4.39)
Drowning	1.90(1.20,2.66)	2.11(1.33,2.96)	2.85(1.79,3.99)	4.83(3.05,6.75)
Poisoning	−0.53(−1.45,0.13)	−0.59(−1.59,0.14)	−0.79(−2.16,0.19)	−1.37(−3.80,0.32)
Suffocation	−0.35(−0.56, −0.14)	−0.39(−0.62, −0.15)	−0.53(−0.83, −0.2)	−0.91(−1.45, −0.35)
Mechanical Force	0.33(0.09,0.57)	0.36(0.10,0.63)	0.49(0.14,0.85)	0.84(0.24,1.44)
Other	0.57(0.08,1.00)	0.63(0.08,1.11)	0.86(0.11,1.50)	1.46(0.19,2.54)
**Intentional Injury**	1.73(0.82,2.63)	1.92(0.91,2.91)	2.59(1.22,3.92)	4.41(2.09,6.68)
Male	2.32(1.08,3.64)	2.57(1.19,4.04)	3.46(1.61,5.44)	5.90(2.75,9.26)
Female	1.04(0.44,1.69)	1.15(0.49,1.88)	1.56(0.66,2.53)	2.66(1.12,4.30)
Age 0–4(y)	0.62(−0.22,1.21)	0.70(−0.24,1.37)	0.94(−0.33,1.87)	1.58(−0.57,3.08)
Age 5–14(y)	0.12(−0.56,0.72)	0.13(−0.61,0.80)	0.18(−0.83,1.09)	0.31(−1.44,1.82)
Age 15–49(y)	1.68(0.90,2.47)	1.86(1.00,2.74)	2.51(1.35,3.70)	4.27(2.30,6.28)
Age 50–69(y)	2.06(0.00,3.98)	2.28(0.00,4.42)	3.08(0.00,5.95)	5.26(0.00,10.12)
Age ≥ 70(y)	7.24(2.57,12.4)	8.02(2.84,13.74)	10.8(3.83,18.52)	18.44(6.56,31.52)
Suicide	1.39(0.69,2.06)	1.54(0.77,2.29)	2.07(1.03,3.08)	3.53(1.77,5.25)
Assault	0.42(0.14,0.73)	0.47(0.16,0.81)	0.63(0.21,1.1)	1.07(0.36,1.85)

### Future injury mortality burden by sex and age

The mortality burden of total injury caused by temperature changes for male is much higher than that of female, so do for mechanism-specific injury such as traffic injury, drowning, and suicide. This may partially be explained by that male is more likely to engage in high-risk work outdoor. For instance, a national survey in China found that male works outdoor for an average of 267 minutes per day, which is higher than the 239 minutes for female^46^. The data from United States also reported similar results^47^. Sex differences in risk-taking may be another important factor. Previous studies have found that risk-taking is positively associated with injury^48^, and that male are more inclined to risk-taking behavior than female, due to the sex disparity of temperament and internalization level^49,50^. In addition, lifestyle may also play a role. For example, the male exceeds female in terms of frequency and quantity of alcohol consumption^51^, and high ambient temperatures may promote this behavior.

For different age groups, the number of deaths from temperature-related injury in the population over 5 years old will rise from the 2060 s to the 2090 s under RCP4.5 and RCP8.5 scenarios (Supplementary Table 3). The age group difference may also be related to exposure and mortality rates to a certain extent^2,46^. However, for each injury, age-specific disparity of injury mortality burden in the future varies. For instance, adults aged 15–49 years have greater temperature-related drowning mortality rate, while the old adults over 75 years have higher transport injury and suicide mortality rate, and children aged 0–4 years and 5–14 years have heavier temperature-related burden in suffocation and poisoning mortality (Supplementary Fig. 5 and Supplementary Fig. 6). This diversity has also been found in a previous study^45^. It is well known that older adults are more susceptible to some temperature-related injuries due to ageing^52^, while the inexperience and lack of awareness were important factors for young populations^53,54^.

### Spatial distribution of future injury mortality burden

Geographically, under RCP4.5 scenario, we observed that the temperature-related injury mortality rates increased from the 2060 s to the 2090 s across China, especially in western China and central China with the highest in Hubei (6.76 in the 2060 s vs. 7.11 in the 2090 s per 100,000). Under RCP8.5 scenario, the corresponding rates are much bigger, and Yunnan is the province with the largest injury mortality burden (10.61 per 100,000 in the 2060 s and 18.16 per 100,000 in the 2090 s) (Supplementary Table 4). Our findings are similar to a previous study on non-accidental death^55^. This may partially be explained by that Western China will be one of the regions with the largest temperature increase in China under future RCP scenarios (Supplementary Fig. 4); and another reason is that higher injury mortality rate is observed in the western and central China^2,56^ (Supplementary Table 5). In addition, the spatial disparity of injury mortality burden may be associated with social vulnerability. In the present study, we found that future injury mortality rates due to temperature change will increase in the provinces with low disposable income per capita and high illiterate rate (Supplementary Table 6). Low-income areas and high illiterate areas are usually accompanied by imperfect environments and low levels of safety awareness^57,58^, which may lead to a higher burden of temperature-related injury mortality. Especially for intentional injury, income inequality may cause higher crime and suicide rates^59,60^, and temperatures may exacerbate this situation. It is worth noting that previous studies on the vulnerability of high temperature exposure mainly focused on non-accidental deaths^61,62^, and the vulnerability factors and mechanisms of temperature-related injuries have not yet been fully elucidated. However, if we used the projected number of deaths as an assessment indicator, we found that injury deaths resulted from temperature change are greater in central or southern China (Supplementary Fig. 7 and Supplementary Table 7), and this is mainly due to the dense population in these areas.

### Sensitivity analysis

We conducted several sensitivity analyses. First, we tested the robustness of the associations of temperature with injury deaths by adjusting for air pollutants, changing maximum lag days, using moving average daily mean temperature, daily maximum and minimum temperature, and found that the associations were stable (Supplementary Table 8). Second, we tested the influence of five General Circulation Models (GCMs) on projected number and mortality rates of injury attributable to temperature change in the future and observed stable results, except for the GFDL-ESM2M model (Supplementary Table 9 and Supplementary Table 10). The difference in injury deaths may due to the differences of future temperature predictions by five GCMs. Finally, we conducted sensitivity analysis based on future population projected by five Shared Socioeconomic Pathways (SSP) scenarios. The projected numbers of population under different SSP scenarios in the future take into account the variables of fertility, mortality, population mobility and education level (Supplementary Table 11)^63^. We found that temperature-related injury deaths were the lowest under the SSP4 scenario, while the highest was observed under the SSP3 scenario. The projected numbers under all SSP population scenarios are lower than that based on the constant population in the 2017 (Supplementary Table 9), which could be mainly explained by the reduced numbers of projected population under all SSP scenarios.

### Strengths and limitations

This study has several strengths. First, we applied a large samples of injury deaths in China to comprehensively explore the association between temperature and total injury mortality and mechanism-specific injury mortality. Second, we projected the burden of injury-related mortality caused by future temperature changes at national and provincial levels. Our findings may be helpful to inform policy development to better adapt to climate change. There are also several limitations that should be concerned. First, although we have included a large sample from six provinces in China, this only accounts for around 20% population in China and does not fully cover all climatic zones, economic levels and ethnic groups, and the representativeness of the sample is still limited. Therefore, caution should be exercised when extrapolating the exposure-response relationships. Second, in the estimation of injury mortality burden attributed to future temperature changes, we used exposure-response relationships and injury mortality based on historical data rather than future data due to data inaccessibility. Future economic development and adaptation capacity may also vary considerably, which make temperature-injury relationship and injury mortality change dramatically, and this may introduce uncertainty of our assessment. Third, we applied the pooled exposure-response relationship to each province in the assessment, and ignored the spatial heterogeneity of each province, especially as China is a vast country, which may lead to biases in spatial distribution. Fourth, the latest projected temperature data based on CMIP6 was not used in this study, but a previous study has shown that CMIP6 may have lower predictive performance for historical temperature than CMIP5 in terms of simulating spatial variability^64^. Fifth, this study did not include injury death from natural disasters, but the frequency of natural disasters was expected to increase as temperatures rise.

In conclusion, the increase in daily mean temperature is associated with greater risk of injury death, and future temperature rise driven by climate change may lead to an increase in the mortality burden of injury in China, particularly traffic injury, drowning, and suicide. Our findings provide evidence on the role of temperature on injury, which may better inform the allocation of resources, the development of climate change response action plans, and the establishment of early warning systems for temperature-related health risk in the context of global warming.

## Methods

### Study area

Six provinces in China were included in this study, namely Guangdong Province in Southern China, Hunan Province in Central China, Zhejiang Province in Eastern China, Yunnan Province in Southwestern China, Tibet Autonomous Region in Western China, and Jilin Province in Northern China (Supplementary Fig. 1). The six provinces cover more than 320 million people in 2019, accounting for 22.9% of population in China^56^.

### Injury death data

We collected all injury death records from Disease Surveillance Points System (DSPS) in Guangdong (2013-2018), Hunan (2013-2018), Zhejiang (2013-2018), Yunnan (2013-2018), Tibet (2013-2019), and Jilin (2013-2018). Individual information including date of death, cause of death, residential address (sub-district level), sex, and age for each case was recorded. Cause of death were categorized using the International Classification of Diseases tenth Revision (ICD-10), of which deaths from injury were coded by V00-Y98. We first divided total injury into unintentional injury (V00-X59), intentional injury(X60-Y09), and other injury (Y10-Y98) by intention, and further divided them according to the mechanism, including transport injury (V00-V99), fall (W00-W19), mechanical force(W20-W64), drowning (W65-W74), suffocation (W75-W84), poisoning (X40-X49), other unintentional injury (W85-W99, X50-X59), suicide (X60-X84), and assault(X85-Y09) (Supplementary Table 12). The injury deaths were also grouped by sex (male and female) and age (0–4 years, 5–14 years, 15–49 years, 50–69 years, and 70 years and over). The injury mortality rates in 2017 for China and each Province were collected from the Global Burden of Diseases, Injuries, and Risk Factors Study (GBD)^2,65^, including total injury rates (nationwide, provincial-level, sex, age group) and cause-specific injury rates (Supplementary Table 5).

### Meteorological and air pollution data

We collected meteorological data (2013-2019) in 698 monitoring stations across China from the China Meteorological Data Sharing Service System (http://data.cma.cn/). Daily mean temperature (Tm) and relative humidity (Rh) were applied a thin plate smoothing spline function and interpolated to 0.01° × 0.01° (approximately equal to 1 km × 1 km) resolution grids^12,66^. The interpolation function included longitude, latitude and altitude as variables, and the results were verified with well accuracy by a 10-fold cross-validation method (Tm: *R*^2^ = 0.96, root mean squared error = 2.37 °C; Rh: *R*^2^ = 0.81, root mean squared error = 7.70%). Based on the residential address and death time of each case, the daily average temperature and relative humidity for each injury death were extracted.

Projected daily temperatures across China for the periods 2010-2019 (2010s), 2060-2069 (2060 s), and 2090-2099 (2090 s) were obtained from the dataset of Coupled Model Intercomparison Project phase 5 (CMIP5)^67^, which is developed by Inter-Sectoral Impact Model Intercomparison Project (ISI-MIP, https://www.isimip.org)^68^. This study focused on two representative concentration pathways (RCP) 4.5 and 8.5, which were also selected by several previous studies^55,69,70^. The RCP4.5 assumes that global greenhouse gas emissions will peak around 2040 and then decline, representing a relatively good scenario; The RCP8.5 assumes that emissions will continue to increase in the absence of climate change mitigation policies, representing a bad scenario. We obtained the daily mean temperature for the above three decades under each RCP scenario in five General Circulation Models (GCMs), including GFDL-ESM2M, HadGEM2-ES, IPSL-CM5A-LR, MIROC-ESM-CHEM, and NorESM1-M, which provide representative projections of future climate across the CMIP5 models. All the daily mean temperatures were bias-corrected and downscaled at a 0.5° × 0.5° spatial resolution^71^. Finally, we extracted the daily average temperature across the whole country and province.

The monitoring station data of daily mass concentration of ambient air pollutants in the six study Provinces during 2013-2019 were collected from the National Urban Air Quality Real-time Publishing Platform (https://air.cnemc.cn:18007/), including particulate matter with an aerodynamic diameter of 10 μm or less (PM_10_), particulate matter with an aerodynamic diameter of 2.5 μm or less (PM_2.5_), Nitrogen dioxide (NO_2_), Sulfur dioxide (SO_2_), and carbon monoxide (CO). A random forest model-based land use regression approach, which is consistent with Liu et al.^12,72^, was conducted in each province to assess exposure to the above air pollutants for each injury death. This approach incorporated 14 predictor variables: latitude, longitude, altitude, daily temperature, daily relative humidity, daily precipitation, daily wind speed, population density, road density, gross domestic product (GDP) per capita, and the proportion of the four land use types (water area, forest land, cropland, and living land). The assessment process was shown in Supplementary Fig. 8. Each air pollutant has been validated with well predictive accuracy (Supplementary Table 13).

### Other data

Data on population, employment, education, and income of each province in 2017 were collected from the National Statistical Yearbook. The population density data in 2015 were obtained from GeoData Institute in University of Southampton (http://www.worldpop.org.uk), and the geographic information system (GIS) covariates (geographic map, road density, land use data and GDP per capita) were obtained from the Data Center for Resources and Environmental Sciences (DCRES, http://www.resdc.cn/). The future populations under five Shared Socioeconomic Pathways (SSPs, SSP1-SSP5) were collected from International Institute for Applied Systems Analysis^63^ (https://iiasa.ac.at)(Supplementary Fig. 9 and Supplementary Table 11).

### Estimating the association of temperature with injury deaths

We constructed a time-stratified case-crossover design and applied conditional logistic regressions to evaluate the association between injury death and ambient temperature^73,74^. For each injury death, exposure to daily mean temperature on the day of death occurred (case day) was compared to the exposure on the same days of the week in the same calendar month (control days). In other words, each injury death had 3 or 4 self-controls. This method of matching case and controls can effectively control a series of confounding, such as long-term and seasonal trends, influence of day-of-week, sex, age, economic conditions, and lifestyle^75,76^.

The relationship between ambient temperature and injury mortality estimated by a two-stage analysis. In the first stage, we used the conditional logistic regression to fit the province-specific relationship^74,77^. The formula is as follow:

1 $${{{{{\rm{Log}}}}}}\left(P({Case}=1\,)\right)={Strata}+{cb}\left({tem}\right)+{ns}\left({rh}\right)+{ns}\left({{PM}}_{2.5}\right)+{Holiday}+\alpha$$

where Log*(P(Case* = *1))* refers to the conditional probability of being a case of injury death; *Strata* is an indicator variable of stratum, and each stratum contains 1 case of injury death (case = 1) and 3 or 4 self-controls (case = 0); *cb(tem)* represents a matrix generated by a cross-basis function for daily temperature exposure, which was used to model the nonlinear or liner relationship on exposure-response dimension and lag-response dimension^78^. Since the onset of injury death is usually more rapid than chronic diseases^55,79^, we applied 1 day as maximum lag period for lag-response dimension based on our initial analyses (Supplementary Fig. 10) and previous study^80^. We first applied a B-spline function with three degrees of freedom (*df*) for the exposure-response dimension. If the result is linear or approximately linear, we would use a linear function to replace the B-spline function. *ns* is a natural cubic spline function, and *rh* and *PM*_*2.5*_ represent the exposure of relative humidity and PM_2.5_ concentration, respectively; *Holiday* refers to a binary variable for control the effect of public holiday; *α* refers the intercept. We then reduced the lag-response dimension in the relationship to calculating the overall effect in each province^81^.

In the second stage, a multivariate meta-analysis was used to examine the pooled effect of temperature on injury death in China^81^. If the temperature-injury death association was considered linear or approximately linear, the result was presented by the indicator of excess risk with 95% confidence interval (CI), which means percentage change of injury death risk for per 1 degree Celsius (°C) increase in daily mean temperature. We further performed stratified analyses by sex, age group (0–4 years, 5–14 years, 15–49 years, 50–69 years and 70+ years), intention, and mechanism.

### Projecting future mortality burden of injury caused by temperature change

Based on the associations between temperature and injury deaths in China, we projected the number of injury deaths attributable to temperature change in the future (compared to the 2010s):

2 $${{AN}}_{g,r}=\sum \left[\frac{{e}^{({T}_{p,g,r,t}-{T}_{h,g,r,t})\times \beta }-1}{{e}^{({T}_{p,g,r,t}-{T}_{h,g,r,t})\times \beta }}\times M\times {pop}\right]$$

where subscript *g* represents the GCMs, and *r* refers RCP scenario; *T*_*p,g,r,t*_ is the projected daily temperature on day *t* under GCM *g* and RCP *r* in the 2060 s or the 2090 s; *T*_*h,g,r,t*_ denotes the projected daily temperature on day *t* under GCM *g* and RCP *r* in 2010s (baseline); *β* refers to the cumulative effect of temperature on injury death; *M* refers to the mean daily mortality rate of injury; *pop* represents the number of population. The projected numbers of injury deaths under different RCP scenarios in the 2060 s and the 2090 s were obtained by averaging those on all GCMs. We further estimated projected number of injury deaths attributable to temperature change in 33 provinces (municipalities, autonomous regions or special administrative regions) across China, which are based on the national pooled effect of temperature on injury death, provincial population and projected daily temperatures. This estimate assumes that climate change adaptation and population are constant and consistent within the period.

We also projected the attributable fraction of injury deaths attributable to temperature change by dividing the projected number by the population. Finally, we used Monte Carlo simulations to obtain the 95% empirical confidence intervals (CIs) from 1000 samples, quantifying the uncertainty in both the relationships of temperature on injury death and the mortality rate of injury. The 95% CIs defined as the 2.5th and 97.5th percentiles of the sample values.

### Meta-regression model

We conducted a meta-regression model to explore the impact of province-level socioeconomic factors on projected rate of injury attributable to temperature change in the future. These province-level variables included proportion of population over 60 years, percentage of illiterate over 15 years, unemployment rate, disposable income per capita and temperature change in the future.

### Sensitivity analyses

To test the robustness of our findings, we performed a series of sensitivity analysis. First, we adjusted air pollutants in the model, including PM_10_, SO_2_, NO_2_, and CO. Then, we changed the maximum lag periods of temperature to 0 and 2 days. Third, we used the moving average of 0–1 day temperature (lag 01) instead of a cross-basis function as the independent variable to evaluate the association between temperature and injury death. Moreover, we also tested the effect of daily maximum and minimum temperature. Finally, we used the five GCMs and projected populations under five SSP scenarios to test the future estimation.

All data were prepared and analyzed using R project software (version 4.0.2). The “dlnm” and “mvmeta” package were used to build the matrix of cross-basis, fit the model, and conduct the meta-analysis, respectively. All figures were made using “ggplot2” package. The results of the statistical tests were two-sided with values of *P* < 0.05 as statistical significance.

### Reporting summary

Further information on research design is available in the Nature Portfolio Reporting Summary linked to this article.

## Supplementary information

Supplementary Information

Reporting Summary

## Data Availability

The original datasets generated or analyzed, or both, during this study are not publicly available because of governance restrictions or the identifiable nature of the data. The raw data for injury deaths, meteorology, GIS covariates and CMIP5 GCMs should be obtained through collaboration with the corresponding author’s team (send requests to mawj@gdiph.org.cn), subject to the approval of the data management and all authors, which is determined within 12 weeks. Raw data on future population, population density, provincial characteristics and air pollution are available directly at https://github.com/Hzeros/tm-injury. The summary statistics and descriptive tables in this study are provided in the Supplementary Information.

## Code availability

The code that supports the findings of this study is available upon request (send requests to mawj@gdiph.org.cn).
