# Supplementary material for: Temperature-related mortality in China from specific injury
Source: Nat Commun. 2023 Jan 3;14:37. doi: 10.1038/s41467-022-35462-4 (PMC9810693; doi:10.1038/s41467-022-35462-4)
Supplement: Supplementary file 1 — Supplementary Information [file 41467_2022_35462_MOESM1_ESM.pdf]

Supplementary Information

Temperature-related mortality in China from specific injury

Jianxiong Hu[1#], Guanhao He[2#], Ruilin Meng[3#], Weiwei Gong[4], Zhoupeng Ren[5], Heng Shi[6], Ziqiang Lin[2], Tao Liu[2], Fangfang Zeng[2], Peng Yin[7], Guoxia Bai[6], Mingfang Qin[8], Zhulin Hou[9], Xiaomei Dong[2], Chunliang Zhou[10], Zhuoma Pingcuo[6], Yize Xiao[8], Min Yu[4], Biao Huang[9], Xiaojun Xu[3], Lifeng Lin[3], Jianpeng Xiao[1], Jieming Zhong[4], Donghui Jin[10], Qinglong Zhao[9], Yajie Li[6], Cangjue Gama[6], Yiqing Xu[10], Lingshuang Lv[10], Weilin Zeng[1], Xing Li[1], Liying Luo[1], Maigeng Zhou[7], Cunrui Huang[11], Wenjun Ma[2*]

[1] Guangdong Provincial Institute of Public Health, Guangdong Provincial Center for Disease Control and Prevention, Guangzhou 511430, China

[2] Department of Public Health and Preventive Medicine, School of Medicine, Jinan University, Guangzhou 511443, China

[3] Guangdong Provincial Center for Disease Control and Prevention, Guangzhou 511430, China

[4] Zhejiang Provincial Center for Disease Control and Prevention, Hangzhou 310009, China

[5] State Key Laboratory of Resources and Environmental Information System, Institute of Geographic Sciences and Natural Resources Research, Chinese Academy of Sciences, Beijing 100101, China

[6] Tibet Autonomous Region Center for Disease Control and Prevention, Lhasa 850002, China

[7] The National Center for Chronic and Noncommunicable Disease Control and Prevention, Beijing 100050, China

[8] Yunnan Provincial Center for Disease Control and Prevention, Kunming 650034, China

[9] Jilin Provincial Center for Disease Control and Prevention, Changchun 130062, China

[10] Hunan Provincial Center for Disease Control and Prevention, Changsha 410005, China

[11] Vanke School of Public Health, Tsinghua University, Beijing 100084, China

[#]These authors contributed equally to this work

[*]Correspondence author: Wenjun Ma, Prof., Department of Public Health and Preventive Medicine, School of Medicine, Jinan University, No. 601, Huangpu Road, Tianhe District, Guangzhou, 510632, China. E-mail mawj@gdiph.org.cn.

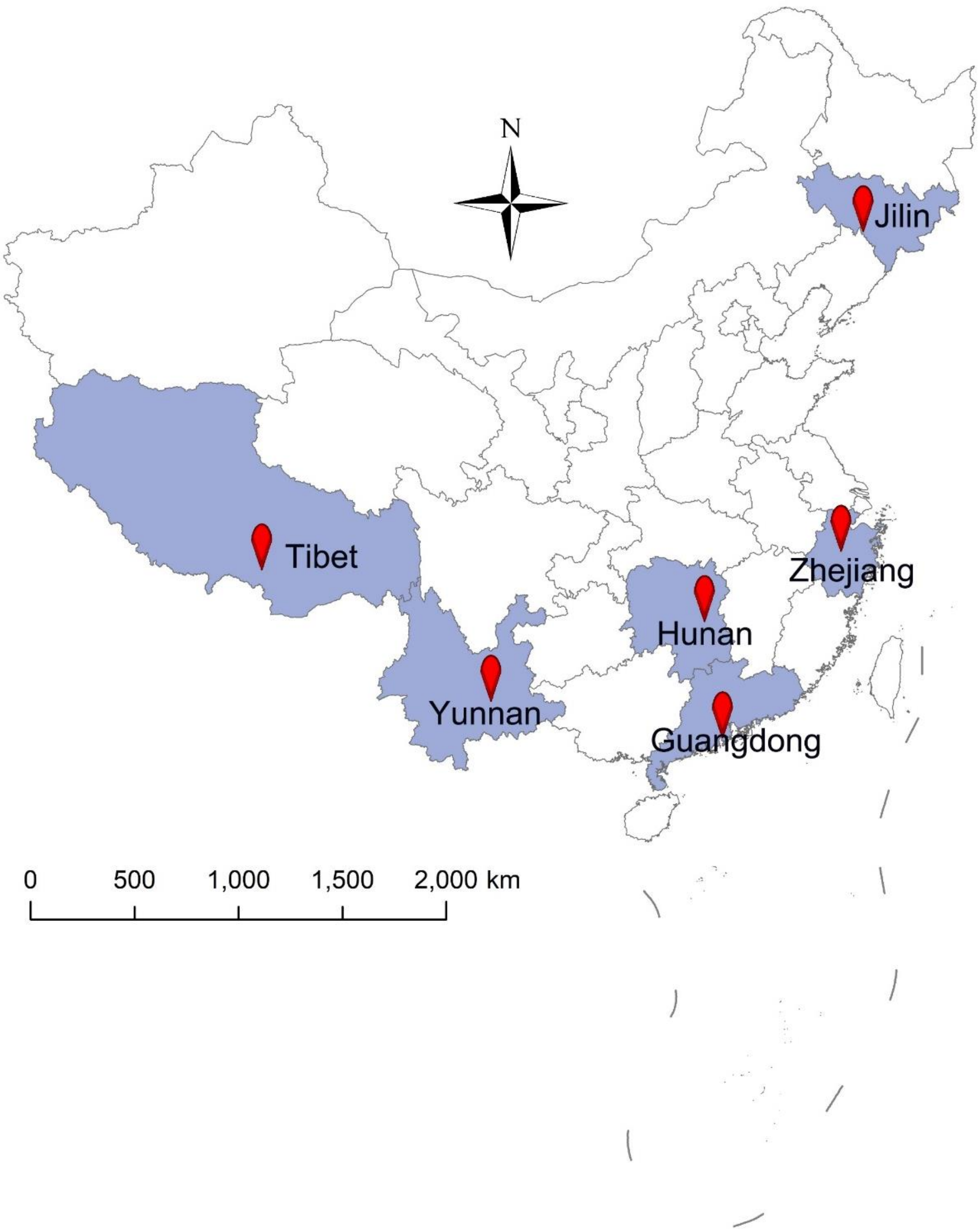

Figure S1. The geographical distribution of studied provinces in China

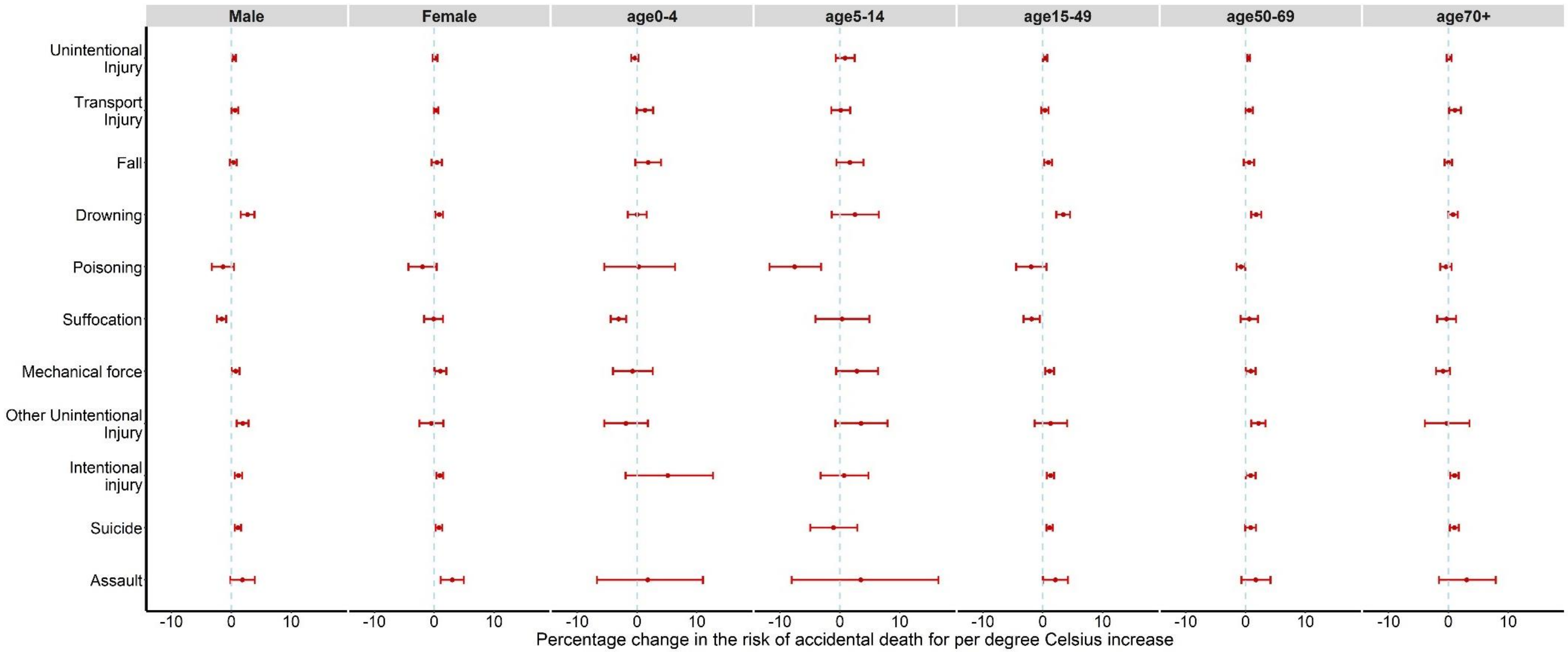

Figure S2. Cumulative excess risk(%) of mechanism-specific injury death for each 1℃ increase in daily mean temperature, by sex and age. The red dots represent the cumulative excess risk of injury death over lag 0-1 days. The horizontal red line represents the 95% confidence interval. N=609827 independent samples of injury deaths were used to conduct 77 independent models; the exact sample sizes are shown as Supplementary Table 1.

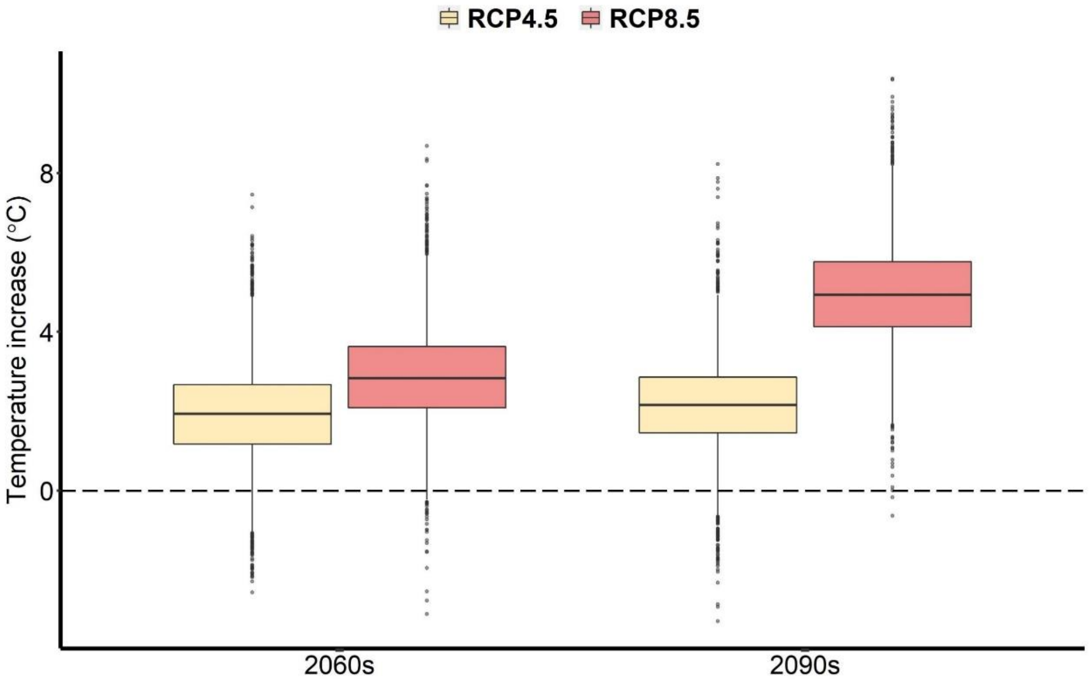

Figure S3. Projected temperature increases in the 2060s and 2090s compared to the 2010s under different RCP4.5 and RCP 8.5 scenario in China. The boxes indicate the first and third quartiles of daily increased temperatures, the black lines within the boxes indicate the median values of daily increased temperatures, and gray dots mark the daily increased temperatures. Each group has N=3652 daily increased temperatures.

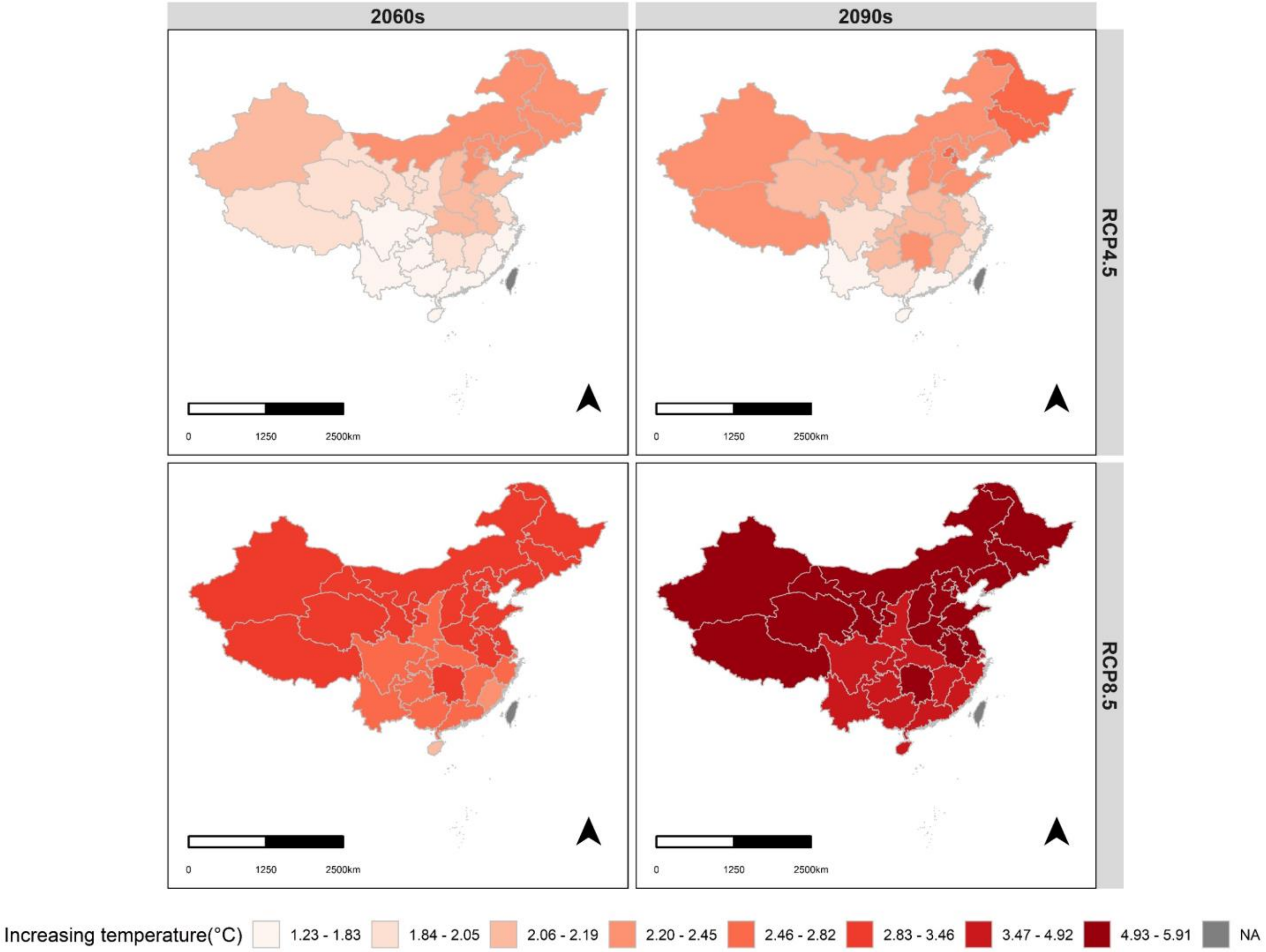

Figure S4. The provincial distribution of projected temperature increases in the 2060s and 2090s compared to the 2010s under different RCP4.5 and RCP 8.5 scenario in China

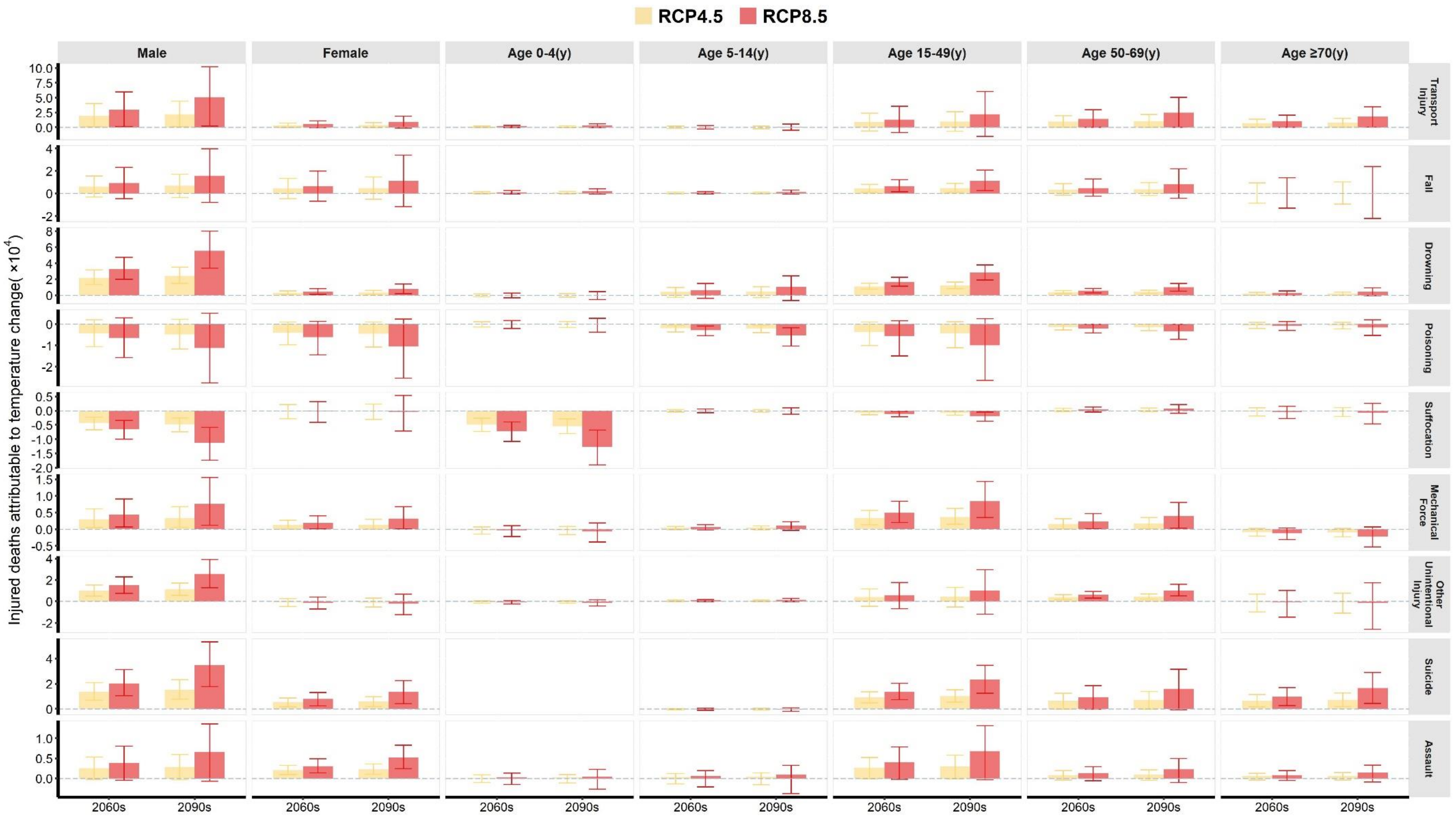

Figure S5. The projected number of injury death attributable to temperature change in the 2060s and 2090s compared to the 2010s under different RCP scenarios in China, by age, sex and mechanism. The bars represent the projected number of injury death attributable to temperature change under different RCP. The vertical solid lines represent the 95% empirical confidence intervals. The horizontal dashed lines denote the reference line (projected number of injury death=0).

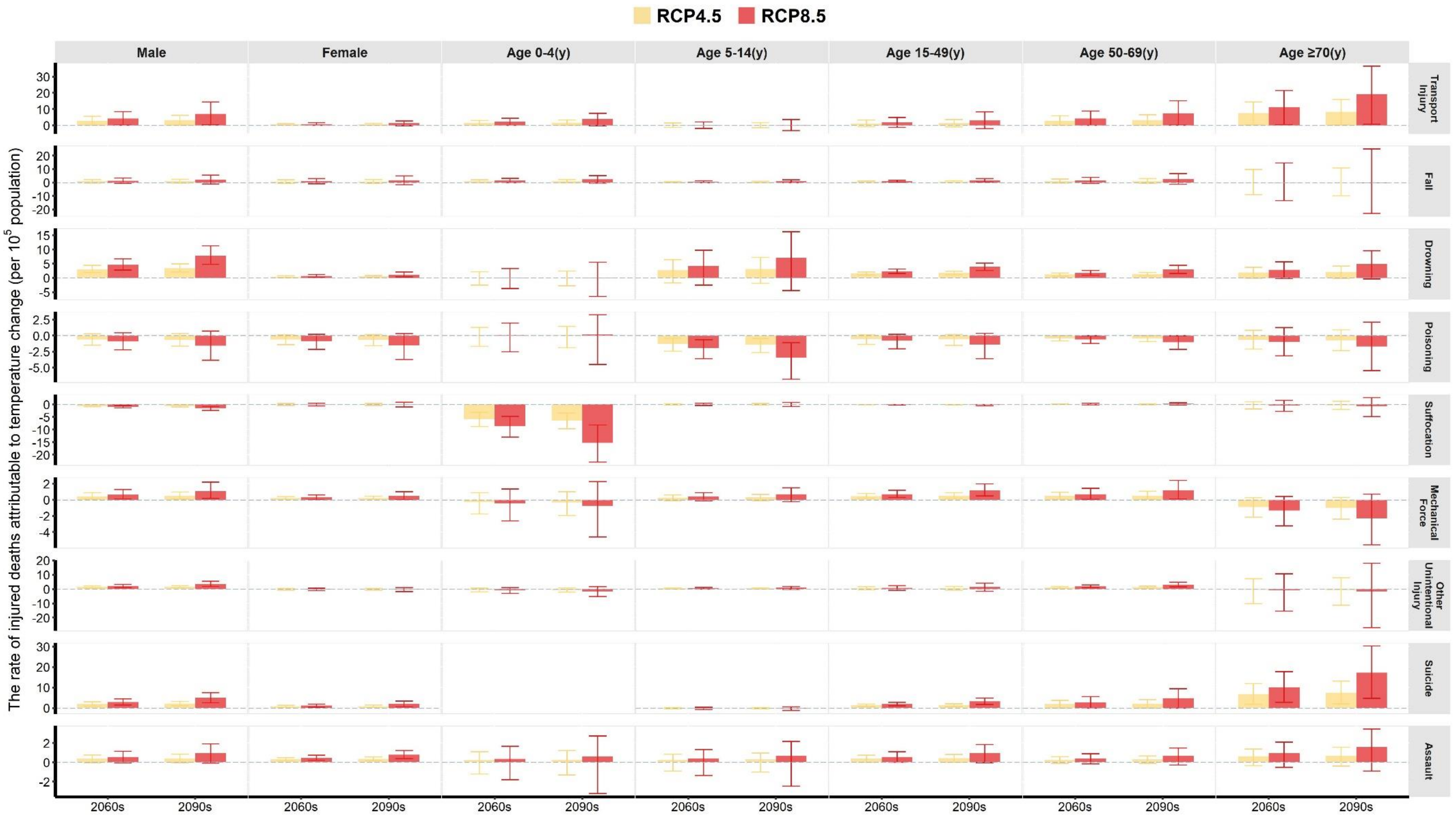

Figure S6. The projected rate (per 100 000) of injury death attributable to temperature change in the 2060s and 2090s compared to the 2010s under different RCP scenarios in China, by age, sex and mechanism. The bars represent the projected rate (per 100 000) of injury death attributable to temperature change under different RCP. The vertical lines represent the 95% empirical confidence intervals. The horizontal dashed lines denote the reference line (projected rate of injury death=0).

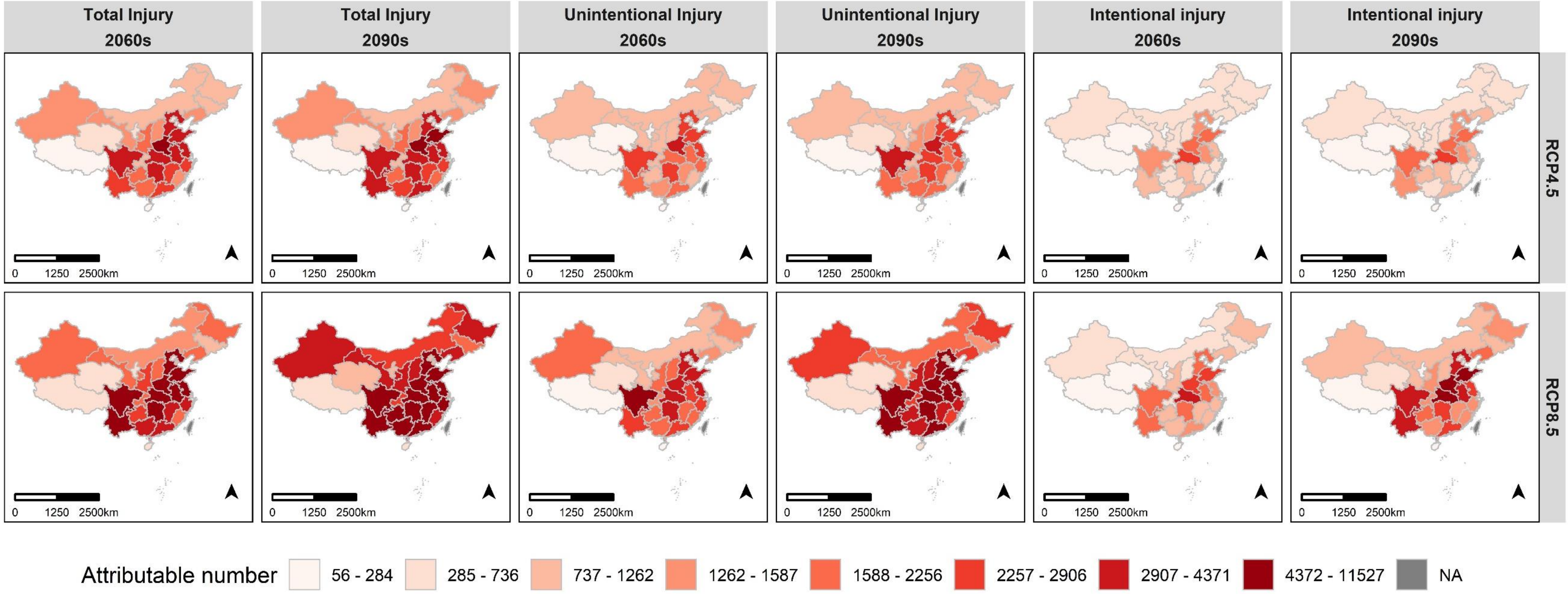

Figure S7. The geographical distribution of projected injury deaths attributable to temperature change in the 2060s and 2090s compared to the 2010s under different RCP scenarios in China

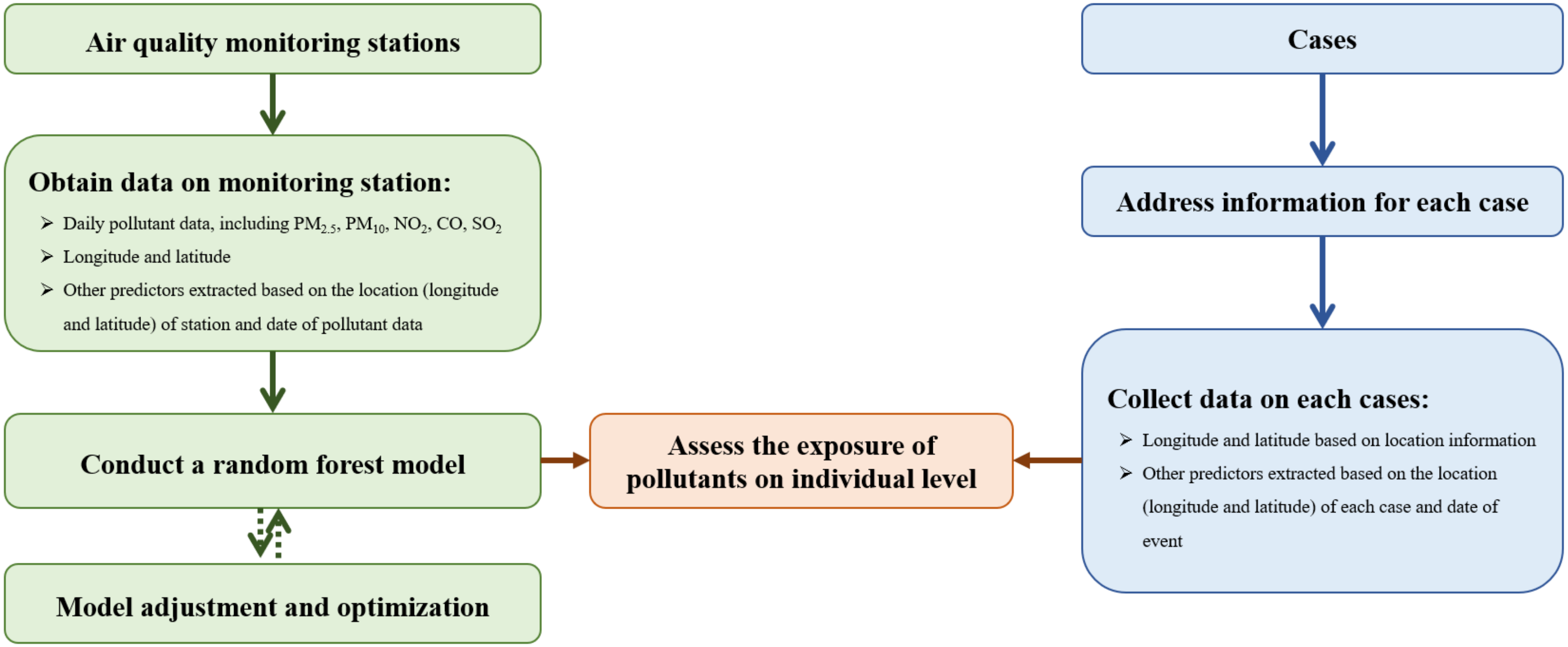

Figure S8. The process of land use regression approach

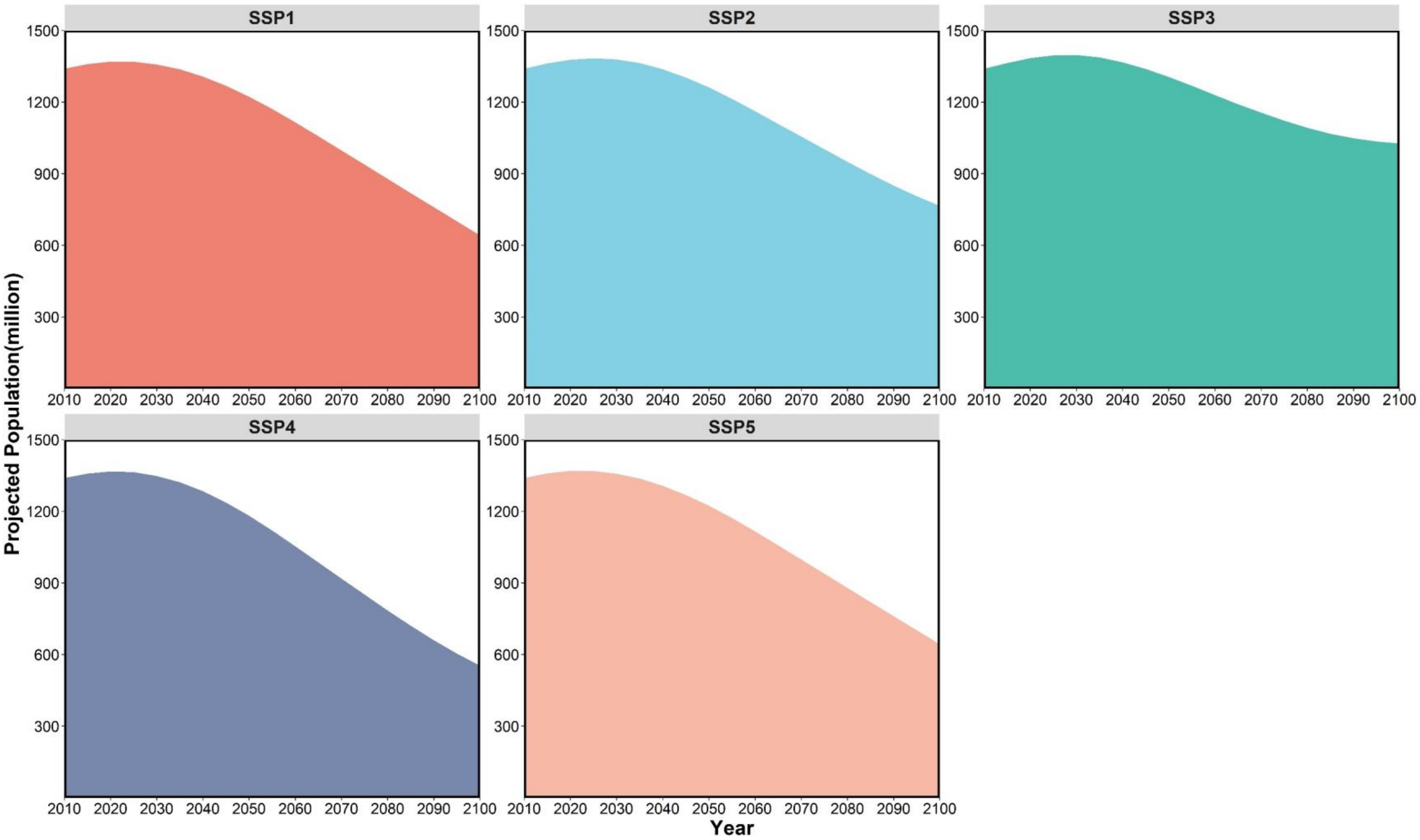

Figure S9. The projected number of populations under five Shared Socioeconomic Pathways in China, 2010-2100

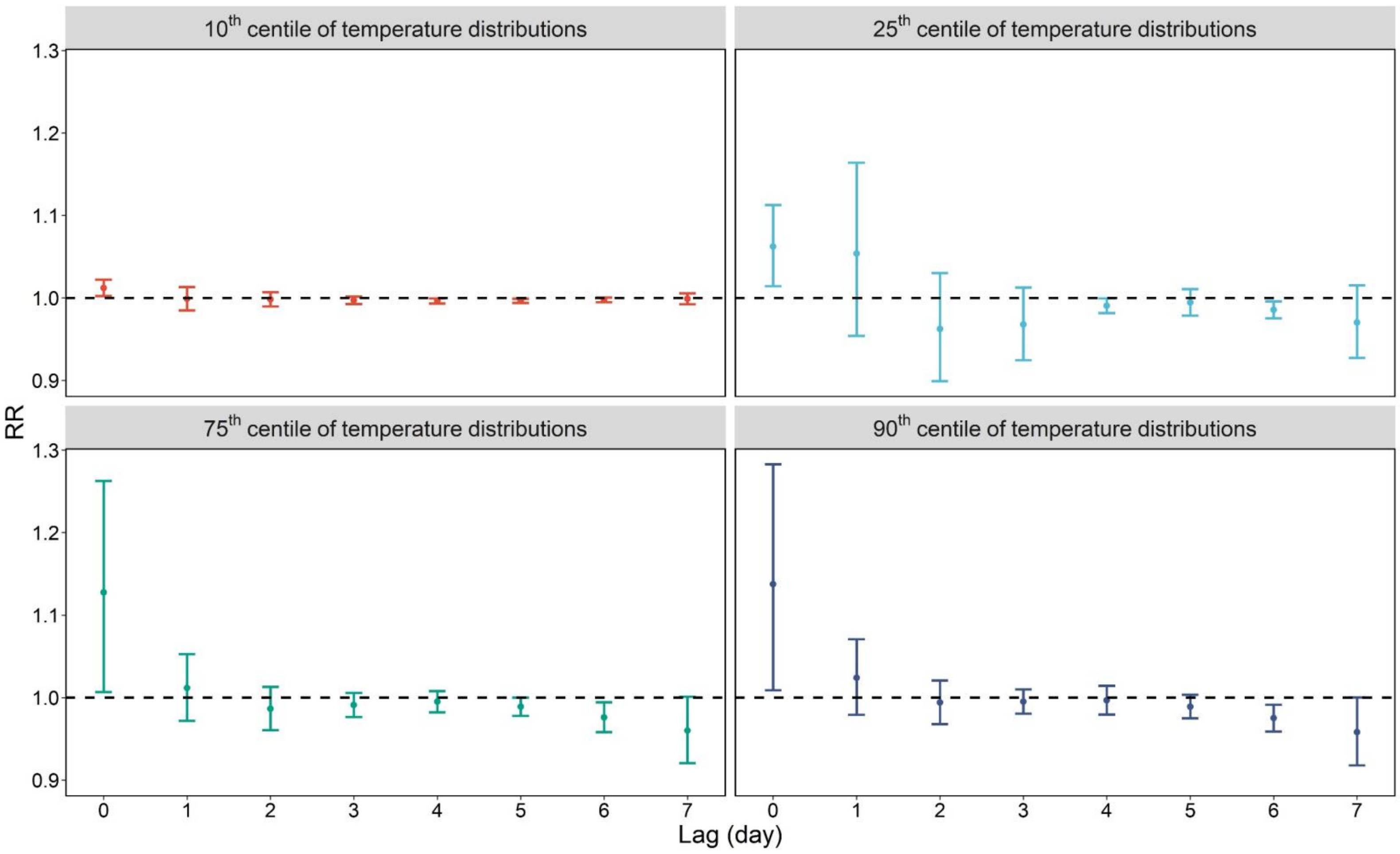

Figure S10. The lag pattern of ambient temperature on total injury deaths for four temperatures (The 10th, 25th, 75th and 90th centile of temperature distributions corresponds to 2.7℃, 8.1℃, 21.8℃ and 24.8℃). The dots represent the relative risk of injury death with different lags at 2.7℃(red), 8.1℃ (light blue), 21.8℃(green) and 24.8℃ (dark blue). The vertical line represents the corresponding 95% confidence interval. The horizontal dashed lines denote the reference line (relative risk=1). All independent samples of injury deaths (N=609827) were incorporated in the models. RR, relative risk.

Table S1. The age and sex characteristics of intention-specific injury deaths in six provinces, China (n, %)

Injury intention	Sex			Age (year)				
	Male	Female	Unknow	0-4	5-14	15-49	50-69	70+
**Unintentional injury**	340596(67.57)	163425(32.42)	19(0.00)	17348(3.44)	15431(3.06)	173304(34.38)	137712(27.32)	160245(31.79)
Transport injury	142105(73.85)	50311(26.15)	8(0.00)	3961(2.06)	4787(2.49)	89918(46.73)	65095(33.83)	28663(14.90)
Fall	88465(56.76)	67381(43.24)	2(0.00)	1900(1.22)	1645(1.06)	24440(15.68)	32121(20.61)	95742(61.43)
Drowning	29888(64.55)	16413(35.45)	0(0.00)	5393(11.65)	6475(13.98)	14065(30.38)	9805(21.18)	10563(22.81)
Poisoning	27523(73.06)	10146(26.93)	2(0.01)	479(1.27)	802(2.13)	17989(47.75)	11784(31.28)	6617(17.57)
Suffocation	13888(66.02)	7147(33.98)	1(0.00)	4340(20.63)	492(2.34)	4104(19.51)	3993(18.98)	8107(38.54)
Mechanical force	22376(78.24)	6222(21.76)	2(0.01)	656(2.29)	697(2.44)	13696(47.89)	9241(32.31)	4310(15.07)
Other unintentional injury	16351(73.79)	5805(26.20)	4(0.02)	619(2.79)	533(2.41)	9092(41.03)	5673(25.60)	6243(28.17)
**Intentional injury**	47248(62.26)	28643(37.74)	2(0.00)	191(0.25)	800(1.05)	32345(42.62)	24301(32.02)	18256(24.05)
Suicide	42934(61.84)	26497(38.16)	2(0.00)	10(0.01)	553(0.80)	27926(40.22)	23056(33.21)	17888(25.76)
Assault	4314(66.78)	2146(33.22)	0(0.00)	181(2.80)	247(3.82)	4419(68.41)	1245(19.27)	368(5.70)
**Other**	15857(53.04)	14036(46.95)	1(0.01)	366(1.22)	360(1.20)	6834(22.86)	5501(18.40)	16833(56.31)

Table S2. The environmental exposure factors of injury death cases in six provinces, China

**Injury**	**Temperature (℃)**				**Relative Humidity (%)**				**PM$_{2.5}$(μg/m^3)**			
	**Case group**	**Control group**	**t value**	***P***	**Case group**	**Control group**	**t value**	***P***	**Case group**	**Control group**	**t value**	***P***
**Total injury**	17.92±8.25	17.95±8.24	8.47	<0.001	75.83±12.45	76.08±12.41	-15.14	<0.001	39.49±27.12	39.36±27.48	5.60	<0.001
**Unintentional injury**	17.92±8.25	17.89±7.81	6.60	<0.001	75.89±12.39	76.15±9.14	-16.21	<0.001	39.68±27.32	39.90±22.97	5.61	<0.001
Transport injury	17.83±8.26	17.78±7.85	5.5^{0}	<0.001	75.76±12.52	75.95±9.42	-7.41	<0.001	40.56±28.46	40.97±24.13	0.58	0.56
Fall	17.75±8.08	17.73±7.62	1.87	0.06	76.16±12.22	76.37±8.72	-7.11	<0.001	39.15±25.47	39.17±20.87	5.27	<0.001
Drowning	20.41±7.79	20.22±7.35	11.61	<0.001	76.68±11.51	77.22±8.29	-10.75	<0.001	37.54±25.57	37.71±21.24	1.15	0.25
Poisoning	15.93±8.29	16.07±7.78	-6.99	<0.001	74.58±13.32	74.72±10.25	-2.51	0.01	38.01±27.17	38.42±23.46	1.67	0.10
Suffocation	16.66±8.29	16.76±7.79	-4.07	<0.001	76.00±12.53	76.23±9.05	-2.79	0.01	41.54±28.37	41.65±24.15	2.26	0.02
Mechanical force	17.64±8.27	17.55±7.88	3.78	<0.001	75.66±12.44	75.96±9.39	-4.52	<0.001	42.15±30.77	42.38±25.92	1.77	0.08
Other unintentional injury	19.74±8.78	19.64±8.17	4.02	<0.001	75.77±12.18	76.57±8.75	-10.64	<0.001	38.18±27.11	38.15±23.33	3.51	<0.001
**Intentional injury**	17.99±8.13	17.90±7.77	7.18	<0.001	75.25±12.95	75.28±10.08	-0.77	0.44	37.93±26.39	38.23±22.20	0.40	0.69
Suicide	17.95±8.13	17.87±7.77	6.18	<0.001	75.25±12.97	75.26±10.10	-0.36	0.72	37.97±26.52	38.23±22.24	0.70	0.48
Assault	18.39±8.06	18.20±7.69	4.39	<0.001	75.30±12.77	75.50±9.87	-1.47	0.14	37.46±24.91	38.18±21.78	-0.95	0.34
**Other**	17.62±8.44	17.63±7.95	-0.09	0.93	76.31±12.27	76.35±8.74	-0.61	0.54	40.19±25.35	40.31±20.75	1.53	0.13

Note: Group mean exposure is expressed as (mean ± standard deviation); t-value were calculated by paired t-test; all the statistical tests were two-sided with values of $P<0.05$ as statistical significance.

Table S2. The environmental exposure factors of injury death cases in six provinces, China (continuous)

Injury	PM_{10} (μg/m³)				SO_2 (μg/m³)				NO_2(μg/m³)				CO (mg/m³)			
	**Case group**	**Control group**	**t value**	***P***	**Case group**	**Control group**	**t value**	***P***	**Case group**	**Control group**	**t value**	***P***	**Case group**	**Control group**	**t value**	***P***
**Total injury**	63.30±36.41	63.04±36.68	8.11	<0.001	15.75±9.89	15.68±9.86	9.45	<0.001	25.20±14.84	25.10±14.76	8.84	<0.001	0.91±0.32	0.90±0.32	1.46	0.14
**Unintentional injury**	63.59±36.62	63.71±29.87	8.03	<0.001	15.76±9.91	15.78±8.53	9.65	<0.001	15.76±9.91	15.78±8.53	8.22	<0.001	0.91±0.32	0.91±0.28	1.09	0.28
Transport injury	64.77±37.80	65.10±31.12	2.48	0.01	16.18±10.30	16.23±8.88	4.29	<0.001	16.18±10.30	16.23±8.88	5.23	<0.001	0.92±0.33	0.92±0.29	0.99	0.32
Fall	62.95±34.75	62.83±27.61	6.27	<0.001	14.99±9.18	14.96±7.84	7.35	<0.001	14.99±9.18	14.96±7.84	5.41	<0.001	0.89±0.30	0.89±0.26	2.41	0.02
Drowning	60.80±34.79	60.88±28.27	1.80	0.07	15.00±9.00	14.98±7.71	3.02	<0.001	15.00±9.00	14.98±7.71	-1.83	0.07	0.88±0.31	0.88±0.26	-3.42	<0.001
Poisoning	61.17±36.31	61.55±29.89	2.11	0.03	16.85±10.86	16.98±9.33	1.56	0.12	16.85±10.86	16.98±9.33	1.84	0.07	0.93±0.36	0.94±0.32	0.32	0.75
Suffocation	65.58±37.63	65.77±31.34	1.60	0.11	15.86±10.11	15.87±8.72	2.70**	0.01	15.86±10.11	15.87±8.72	3.38	<0.001	0.92±0.32	0.92±0.28	1.82	0.07
Mechanical force	67.06±40.57	67.13±33.33	2.50	0.01	17.03±10.75	17.02±9.24	3.18	<0.001	17.03±10.75	17.02±9.24	3.14	<0.001	0.94±0.34	0.95±0.30	-0.63	0.53
Other unintentional injury	61.37±35.97	60.97±29.84	4.98	<0.001	15.43±9.70	15.50±8.50	1.91	0.06	15.43±9.70	15.50±8.50	2.00	0.05	0.88±0.31	0.89±0.28	-0.79	0.43
**Intentional injury**	61.16±35.51	61.45±28.76	1.07	0.28	15.90±9.99	16.00±8.75	0.79	0.43	15.90±9.99	16.00±8.75	1.19	0.23	0.91±0.33	0.92±0.29	-0.04	0.97
Suicide	61.22±35.67	61.48±28.80	1.17	0.24	15.86±9.97	15.94±8.69	1.17	0.24	15.86±9.97	15.94±8.69	1.62	0.11	0.91±0.33	0.91±0.29	0.38	0.71
Assault	60.49±33.70	61.18±28.32	-0.19	0.85	16.33±10.16	16.59±9.38	-1.06	0.29	16.33±10.16	16.59±9.38	-1.12	0.26	0.92±0.34	0.93±0.31	-1.32	0.19
**Other**	63.84±34.89	63.89±27.62	1.84	0.07	15.21±9.27	15.25±7.99	1.85	0.06	15.21±9.27	15.25±7.99	3.80	<0.001	0.88±0.30	0.88±0.25	2.29	0.02

Note: Group mean exposure is expressed as (mean ± standard deviation); t-value were calculated by paired t-test; all the statistical tests were two-sided with values of $P<0.05$ as statistical significance.

Table S3. The projected number of injury deaths attributable to temperature change in the 2060s and 2090s compared to the 2010s under different RCP scenarios in China

	RCP 4.5		RCP8.5	
	2060s	2090s	2060s	2090s
**Total Injury**	61348(14731,106828)	67895(16294,118285)	91480(21956,159371)	156586(37654,272316)
Male	49134(21021,79878)	54381(23257,88444)	73271(31337,119161)	125384(53705,203627)
Female	11893(-4109,27942)	13159(-4544,30932)	17731(-6123,41680)	30378(-10515,71269)
Age 0-4(y)	-1309(-4624,1552)	-1447(-5108,1717)	-1950(-6885,2314)	-3352(-11871,3964)
Age 5-14(y)	4148(-1871,9815)	4594(-2068,10894)	6189(-2787,14679)	10570(-4793,24891)
Age 15-49(y)	26388(7773,43311)	29205(8598,47955)	39350(11586,64610)	67348(19866,110413)
Age 50-69(y)	21587(11149,31929)	23893(12336,35351)	32192(16622,47629)	55087(28476,81409)
Age≥70(y)	8426(-5945,23383)	9322(-6573,25881)	12560(-8858,34872)	21529(-15213,59667)
**Unintentional Injury**	40736(5897,75398)	45077(6522,83473)	60737(8789,112465)	104014(15077,192266)
Male	35239(10409,59372)	38997(11514,65731)	52544(15515,88576)	89953(26605,151380)
Female	5096(-7530,17742)	5637(-8325,19637)	7595(-11219,26458)	13023(-19274,45272)
Age 0-4(y)	-1814(-4797,1298)	-2006(-5298,1436)	-2703(-7143,1934)	-4647(-12319,3315)
Age 5-14(y)	3523(-2563,9505)	3901(-2832,10550)	5256(-3817,14215)	8981(-6573,24107)
Age 15-49(y)	16166(930,31504)	17888(1029,34876)	24102(1386,46989)	41278(2379,80345)
Age 50-69(y)	15382(8657,22311)	17024(9578,24698)	22937(12906,33276)	39260(22116,56911)
Age≥70(y)	3824(-8030,15501)	4230(-8877,17154)	5700(-11963,23113)	9775(-20560,39570)
Transport Injury	25383(3331,45446)	28095(3685,50339)	37854(4965,67820)	64764(8517,115743)
Fall	8841(-5177,23927)	9783(-5723,26498)	13181(-7713,35700)	22575(-13252,60963)
Drowning	26466(16668,37021)	29356(18467,41120)	39551(24880,55412)	67206(42427,93794)
Poisoning	-7415(-20149,1761)	-8184(-22155,1949)	-11036(-29959,2626)	-19094(-52770,4494)
Suffocation	-4914(-7801, -1906)	-5425(-8605, -2106)	-7315(-11607, -2839)	-12636(-20134, -4885)
Mechanical Force	4556(1293,7869)	5044(1430,8721)	6796(1927,11749)	11614(3303,20017)
Other	7965(1043,13905)	8824(1153,15425)	11888(1554,20783)	20282(2665,35299)
**Intentional Injury**	24095(11389,36522)	26690(12605,40494)	35958(16984,54556)	61363(29064,92820)
Male	16498(7676,25900)	18276(8497,28712)	24623(11448,38682)	42005(19585,65846)
Female	7075(2989,11483)	7835(3307,12727)	10556(4456,17145)	18029(7632,29206)
Age 0-4(y)	517(-183,1007)	577(-202,1137)	778(-272,1550)	1306(-471,2557)
Age 5-14(y)	182(-836,1088)	201(-922,1212)	271(-1244,1635)	463(-2170,2747)
Age 15-49(y)	12177(6547,17951)	13492(7249,19911)	18177(9766,26819)	30994(16694,45586)
Age 50-69(y)	6916(-1,13358)	7658(-1,14820)	10317(-1,19957)	17630(-1,33914)
Age≥70(y)	6929(2461,11870)	7674(2723,13158)	10339(3669,17726)	17653(6283,30172)
Suicide	19284(9618,28690)	21358(10645,31803)	28775(14342,42848)	49129(24549,72940)
Assault	5846(1971,10187)	6487(2182,11326)	8741(2940,15271)	14834(5028,25767)

Table S4. The projected rate (per 100 000) of injury deaths attributable to temperature change in the 2060s and 2090s compared to the 2010s under different RCP scenarios in 33 provinces, China

	RCP 4.5		RCP8.5	
	2060s	2090s	2060s	2090s
**Total Injury**				
Beijing	1.86(0.52,3.27)	2.05(0.57,3.61)	2.44(0.71,4.37)	4.17(1.21,7.47)
Tianjin	3.30(1.00,5.74)	3.76(1.14,6.56)	4.44(1.20,7.69)	7.61(2.06,13.14)
Hebei	5.02(1.27,8.44)	5.55(1.40,9.34)	6.74(1.92,11.83)	11.53(3.29,20.21)
Shanxi	3.83(0.99,6.63)	4.22(1.09,7.33)	5.45(1.42,9.46)	9.33(2.43,16.17)
Inner Mongolia	4.24(0.89,7.42)	4.53(0.95,7.95)	5.45(1.26,9.88)	9.33(2.15,16.89)
Liaoning	3.31(0.93,5.85)	3.56(1.00,6.31)	4.30(1.09,7.54)	7.36(1.87,12.89)
Jilin	2.88(0.75,5.05)	3.14(0.82,5.51)	3.73(0.88,6.53)	6.38(1.52,11.17)
Heilongjiang	3.33(0.91,6.07)	3.64(0.99,6.65)	4.16(0.98,7.34)	7.12(1.67,12.55)
Shanghai	2.22(0.46,3.86)	2.23(0.46,3.89)	3.44(1.07,6.01)	5.89(1.84,10.27)
Jiangsu	4.18(1.12,7.48)	4.17(1.12,7.48)	6.02(1.62,10.64)	10.31(2.78,18.19)
Zhejiang	4.03(1.17,7.37)	4.26(1.24,7.80)	6.74(1.80,12.14)	11.53(3.09,20.75)
Anhui	5.10(1.31,8.60)	5.13(1.31,8.64)	7.31(1.92,13.78)	12.52(3.29,23.53)
Fujian	3.69(1.06,6.40)	4.41(1.26,7.66)	7.03(2.05,12.17)	12.03(3.52,20.80)
Jiangxi	5.21(1.34,8.87)	5.87(1.5,10.01)	8.03(2.32,13.81)	13.74(3.97,23.59)
Shandong	4.17(1.04,7.39)	4.44(1.11,7.88)	5.73(1.70,10.11)	9.82(2.91,17.28)
Henan	4.97(1.38,8.84)	5.15(1.44,9.19)	7.03(2.00,12.39)	12.03(3.44,21.17)
Hubei	6.76(1.82,11.95)	7.11(1.91,12.58)	9.61(2.15,17.06)	16.44(3.69,29.16)
Hunan	5.41(1.16,9.63)	6.13(1.31,10.94)	8.17(1.74,14.24)	13.99(2.99,24.32)
Guangdong	2.15(0.62,3.64)	2.72(0.78,4.61)	4.44(1.31,8.02)	7.61(2.25,13.70)
Guangxi	4.08(1.09,7.18)	5.05(1.34,8.92)	7.31(1.90,12.48)	12.52(3.26,21.32)
Hainan	2.43(0.66,4.20)	2.91(0.79,5.05)	5.73(1.49,10.22)	9.82(2.55,17.46)
Chongqing	3.57(0.86,6.21)	4.09(0.99,7.14)	5.73(1.50,10.32)	9.82(2.57,17.64)
Sichuan	4.32(1.31,7.57)	5.07(1.54,8.91)	7.46(1.89,13.00)	12.76(3.24,22.22)
Guizhou	4.59(1.20,8.02)	5.51(1.44,9.66)	7.74(1.80,13.48)	13.25(3.09,23.04)
Yunnan	5.30(1.43,9.54)	6.54(1.77,11.78)	10.61(2.51,18.96)	18.16(4.31,32.39)
Tibet	5.61(1.40,9.87)	6.45(1.61,11.35)	8.03(2.11,13.73)	13.74(3.63,23.46)
Shaanxi	4.35(1.10,7.61)	4.60(1.16,8.06)	6.74(1.61,11.82)	11.53(2.76,20.20)
Gansu	4.87(1.35,8.44)	5.25(1.45,9.11)	7.46(1.84,12.99)	12.76(3.16,22.21)
Qinghai	5.52(1.53,9.54)	6.30(1.74,10.89)	8.75(2.18,15.57)	14.97(3.74,26.60)
Ningxia	5.14(1.37,9.05)	5.53(1.47,9.76)	7.60(2.06,13.60)	13.01(3.54,23.22)
Xinjiang	5.30(1.17,9.31)	6.00(1.32,10.56)	7.31(1.42,13.16)	12.52(2.43,22.47)

Hongkong	1.21(0.24,2.08)	1.50(0.30,2.59)	2.72(0.72,4.94)	4.66(1.23,8.44)
Macao	1.28(0.34,2.26)	1.61(0.43,2.84)	2.87(0.77,5.04)	4.91(1.32,8.62)
**Unintentional Injury**				
Beijing	1.59(0.26,2.94)	1.75(0.28,3.25)	2.08(0.17,4.04)	3.57(0.30,6.90)
Tianjin	2.21(0.33,3.94)	2.51(0.38,4.49)	2.96(0.37,5.48)	5.08(0.64,9.36)
Hebei	3.36(0.04,6.38)	3.71(0.04,7.07)	4.50(0.64,8.56)	7.70(1.10,14.63)
Shanxi	3.27(0.26,6.20)	3.61(0.29,6.84)	4.65(0.61,8.60)	7.96(1.04,14.70)
Inner Mongolia	3.06(0.35,5.78)	3.27(0.37,6.18)	3.93(0.60,7.56)	6.72(1.02,12.92)
Liaoning	2.55(0.43,4.62)	2.74(0.46,4.98)	3.30(0.14,6.29)	5.65(0.25,10.75)
Jilin	2.04(0.19,3.79)	2.21(0.20,4.13)	2.62(0.36,5.07)	4.49(0.61,8.66)
Heilongjiang	2.60(0.26,5.03)	2.84(0.29,5.50)	3.23(0.21,6.18)	5.54(0.35,10.56)
Shanghai	1.70(0.15,3.24)	1.71(0.15,3.26)	2.64(0.36,5.07)	4.52(0.62,8.66)
Jiangsu	2.93(0.32,5.75)	2.93(0.32,5.75)	4.22(0.53,8.07)	7.23(0.92,13.80)
Zhejiang	2.87(0.34,5.53)	3.04(0.36,5.85)	4.79(0.48,9.69)	8.20(0.82,16.58)
Anhui	3.17(0.48,6.18)	3.18(0.48,6.21)	4.53(0.61,8.88)	7.75(1.05,15.18)
Fujian	2.70(0.14,5.23)	3.23(0.17,6.27)	5.14(0.78,10.19)	8.80(1.34,17.41)
Jiangxi	3.24(0.48,6.29)	3.65(0.54,7.10)	4.98(0.59,9.52)	8.54(1.01,16.27)
Shandong	2.69(0.40,5.11)	2.86(0.43,5.44)	3.69(0.40,7.03)	6.31(0.69,12.02)
Henan	3.06(0.19,5.71)	3.18(0.20,5.93)	4.32(0.47,8.32)	7.40(0.81,14.22)
Hubei	3.81(0.26,7.06)	4.00(0.27,7.42)	5.40(0.12,10.56)	9.24(0.21,18.05)
Hunan	3.35(0.40,6.38)	3.79(0.45,7.24)	5.04(0.54,9.71)	8.63(0.93,16.6)
Guangdong	1.42(0.21,2.72)	1.79(0.26,3.45)	2.93(0.30,5.64)	5.01(0.52,9.63)
Guangxi	2.73(0.27,5.04)	3.38(0.33,6.25)	4.88(0.72,9.41)	8.35(1.23,16.09)
Hainan	1.65(0.14,3.22)	1.98(0.17,3.86)	3.91(0.30,7.42)	6.69(0.52,12.68)
Chongqing	3.25(0.33,6.33)	3.73(0.38,7.27)	5.22(0.51,9.87)	8.93(0.88,16.87)
Sichuan	3.37(0.26,6.28)	3.96(0.30,7.39)	5.81(1.01,11.24)	9.96(1.73,19.21)
Guizhou	3.38(0.30,6.33)	4.05(0.35,7.63)	5.67(0.64,11.03)	9.71(1.09,18.86)
Yunnan	3.39(0.4,6.44)	4.19(0.49,7.95)	6.80(1.10,12.96)	11.64(1.89,22.14)
Tibet	4.29(0.29,8.08)	4.93(0.34,9.29)	6.14(0.62,11.73)	10.52(1.07,20.05)
Shaanxi	3.22(0.47,6.00)	3.40(0.50,6.35)	4.97(0.53,9.44)	8.50(0.91,16.14)
Gansu	2.91(0.34,5.50)	3.14(0.36,5.94)	4.45(0.40,8.43)	7.62(0.69,14.41)
Qinghai	4.30(0.36,8.23)	4.90(0.41,9.39)	6.80(0.94,12.87)	11.64(1.61,22.01)
Ningxia	4.09(0.44,7.67)	4.41(0.48,8.27)	6.04(0.75,12.00)	10.34(1.28,20.51)
Xinjiang	4.31(0.16,7.87)	4.87(0.18,8.92)	5.93(0.27,11.20)	10.15(0.46,19.15)
Hongkong	0.61(0.07,1.21)	0.76(0.09,1.50)	1.39(0.14,2.74)	2.37(0.25,4.68)
Macao	0.67(0.08,1.26)	0.83(0.1,1.58)	1.48(0.16,2.80)	3.57(0.30,6.90)

**Intentional Injury**				
Beijing	0.80(0.39,1.28)	0.89(0.44,1.42)	1.08(0.51,1.68)	1.84(0.87,2.87)
Tianjin	1.03(0.52,1.65)	1.18(0.60,1.89)	1.41(0.64,2.23)	2.41(1.10,3.79)
Hebei	1.72(0.84,2.66)	1.90(0.93,2.97)	2.34(1.15,3.84)	4.00(1.96,6.52)
Shanxi	1.31(0.64,2.09)	1.45(0.71,2.32)	1.90(0.90,3.11)	3.24(1.53,5.30)
Inner Mongolia	1.83(0.88,2.78)	1.96(0.94,2.99)	2.39(1.16,3.79)	4.08(1.99,6.44)
Liaoning	1.59(0.80,2.39)	1.72(0.86,2.60)	2.12(1.02,3.25)	3.61(1.74,5.54)
Jilin	1.45(0.71,2.28)	1.59(0.77,2.50)	1.93(0.98,3.04)	3.29(1.67,5.18)
Heilongjiang	1.61(0.77,2.51)	1.77(0.84,2.76)	2.06(0.88,3.22)	3.52(1.50,5.48)
Shanghai	0.74(0.36,1.14)	0.74(0.36,1.15)	1.16(0.57,1.82)	1.97(0.97,3.10)
Jiangsu	1.22(0.61,1.86)	1.22(0.61,1.86)	1.79(0.79,2.77)	3.05(1.35,4.72)
Zhejiang	1.05(0.50,1.59)	1.11(0.53,1.69)	1.78(0.89,2.70)	3.03(1.52,4.59)
Anhui	2.40(1.16,3.68)	2.42(1.17,3.71)	3.50(1.60,5.40)	5.97(2.74,9.19)
Fujian	1.19(0.59,1.83)	1.43(0.70,2.20)	2.31(1.08,3.54)	3.94(1.85,6.03)
Jiangxi	1.32(0.58,2.00)	1.49(0.65,2.27)	2.07(0.98,3.21)	3.54(1.68,5.46)
Shandong	1.93(0.93,3.00)	2.06(0.99,3.21)	2.70(1.23,4.38)	4.60(2.10,7.46)
Henan	1.87(0.95,2.83)	1.95(0.99,2.95)	2.70(1.30,4.23)	4.60(2.23,7.20)
Hubei	3.96(1.94,6.17)	4.17(2.04,6.51)	5.73(2.82,8.78)	9.78(4.82,14.95)
Hunan	1.61(0.84,2.50)	1.84(0.95,2.86)	2.50(1.26,3.87)	4.27(2.16,6.59)
Guangdong	0.73(0.35,1.15)	0.93(0.44,1.46)	1.54(0.77,2.42)	2.63(1.31,4.12)
Guangxi	1.06(0.49,1.73)	1.32(0.61,2.16)	1.95(0.79,3.22)	3.33(1.35,5.49)
Hainan	1.04(0.49,1.62)	1.25(0.58,1.95)	2.46(1.16,3.85)	4.2(1.98,6.56)
Chongqing	1.57(0.77,2.42)	1.81(0.88,2.80)	2.57(1.18,4.01)	4.39(2.02,6.82)
Sichuan	1.63(0.77,2.53)	1.92(0.90,2.98)	2.83(1.45,4.45)	4.83(2.48,7.57)
Guizhou	1.83(0.91,2.88)	2.21(1.10,3.51)	3.16(1.45,4.98)	5.39(2.48,8.48)
Yunnan	2.18(1.08,3.33)	2.70(1.33,4.12)	4.38(2.17,6.78)	7.48(3.71,11.55)
Tibet	1.67(0.61,2.76)	1.92(0.70,3.18)	2.40(1.08,3.92)	4.09(1.84,6.68)
Shaanxi	1.56(0.70,2.40)	1.65(0.74,2.55)	2.45(1.23,3.85)	4.19(2.10,6.56)
Gansu	1.74(0.85,2.71)	1.88(0.92,2.94)	2.70(1.29,4.14)	4.60(2.21,7.06)
Qinghai	2.15(1.05,3.44)	2.46(1.20,3.93)	3.44(1.62,5.49)	5.86(2.77,9.34)
Ningxia	1.48(0.65,2.36)	1.60(0.70,2.55)	2.24(1.06,3.72)	3.82(1.81,6.34)
Xinjiang	1.73(0.80,2.73)	1.96(0.91,3.10)	2.42(1.19,3.90)	4.12(2.04,6.64)
Hongkong	0.98(0.47,1.59)	1.22(0.59,1.98)	2.24(1.02,3.73)	3.82(1.75,6.36)
Macao	1.03(0.51,1.70)	1.29(0.64,2.15)	2.32(0.99,3.77)	3.96(1.69,6.43)

Table S5. The mortality rates of injury and population in 2017 in China, by age, sex, intention, mechanism and province

Type	Mortality rate<br>(Per 100000, 95% uncertainty intervals)	Population (million)
**Total injury deaths**	45.9 (42.9, 47.8)	1390.08
**Sex**		
Male	64.8 (55.9, 73.7)	711.37
Female	29.2 (25.3, 33.1)	678.71
**Age**		
0-4	30.5 (26.9, 34.2)	82.92
5-14	15.0 (13.9, 16.0)	150.56
15-49	36.5 (32.6, 40.4)	725.64
50-69	59.4 (51.8, 67.5)	335.22
70+	198.0 (163.0, 233.0)	95.74
**Injury intention**		
Unintentional injury	37.7 (35.6, 39.8)	1390.08
Transport injury	16.1 (15.4, 16.8)	1390.08
Fall	8.6 (6.7, 9.5)	1390.08
Drowning	5.1 (4.9, 5.3)	1390.08
Poisoning	1.7 (0.9, 2.0)	1390.08
Suffocation	1.4 (1.2, 1.5)	1390.08
Mechanical force	2.1 (1.5, 2.3)	1390.08
Other unintentional injury	2.6 (2.5, 2.7)	1390.08
Intentional injury	8.2 (7.8, 9.1)	1390.08
Suicide	7.2 (6.8, 7.9)	1390.08
Assault	1.0 (0.8, 1.3)	1390.08
**Province**		
Beijing	17 (14 ,19)	21.71
Tianjin	31 (26 ,36)	15.57
Hebei	47 (41 ,54)	75.20
Shanxi	38 (32 ,46)	37.02
Inner Mongolia	38 (32 ,44)	25.29
Liaoning	30 (25 ,36)	43.69
Jilin	26 (21 ,33)	27.17
Heilongjiang	29 (24 ,35)	37.89
Shanghai	24 (21 ,28)	24.18
Jiangsu	42 (36 ,49)	80.29
Zhejiang	47 (38 ,56)	56.57
Anhui	51 (43 ,59)	62.55
Fujian	49 (40 ,58)	39.11
Jiangxi	56 (49 ,64)	46.22
Shandong	40 (34 ,48)	100.06
Henan	49 (41 ,57)	95.59

Hubei	67 (53 ,78)	59.02
Hunan	57 (48 ,66)	68.6
Guangdong	31 (27 ,36)	111.69
Guangxi	51 (43 ,60)	48.85
Hainan	40 (34 ,47)	9.26
Sichuan	40 (33 ,48)	30.75
Guizhou	52 (43 ,61)	83.02
Yunnan	54 (45 ,64)	35.80
Tibet	74 (63 ,86)	48.01
Chongqing	56 (47 ,65)	3.37
Shaanxi	47 (38 ,55)	38.35
Gansu	52 (45 ,60)	26.26
Qinghai	61 (52 ,71)	5.98
Ningxia	53 (45 ,64)	6.82
Xinjiang	51 (43 ,59)	24.45
Hongkong	19 (16 ,22)	11.06
Macao	20 (18 ,23)	0.65

Table S6. The impact of socioeconomic factors on the projected rate of injury deaths attributable to temperature change in the future

		RCP 4.5				RCP 8.5			
		2060s		2090s		2060s		2090s	
		β	*P*	β	*P*	β	*P*	β	*P*
Total injury	Proportion of population over 60 years (%)	0.0434	0.67	0.0067	0.95	-0.0162	0.91	0.0064	0.98
	Percentage of illiterate over 15 years (%)	0.0523	0.12	0.0565	0.12	0.0994	0.05	0.1656	0.07
	Unemployment rate (%)	-0.0787	0.79	-0.0067	0.98	-0.0354	0.93	-0.0842	0.91
	Disposable income per capita (×10^4 Yuan)	-0.6449	<0.01	-0.7203	<0.01	-0.9634	<0.01	-1.6737	<0.01
	Future temperature changes (℃)	0.6130	0.36	0.0669	0.93	-0.1936	0.82	0.0444	0.96
Unintentional Injury	Proportion of population over 60 years (%)	-0.0290	0.65	-0.0543	0.43	-0.0925	0.33	-0.1413	0.40
	Percentage of illiterate over 15 years (%)	0.0399	0.07	0.0443	0.07	0.0727*	0.04	0.1207	0.05
	Unemployment rate (%)	0.1262	0.51	0.17597	0.41	0.2172	0.47	0.3559	0.50
	Disposable income per capita (×10^4 Yuan)	-0.3482*	<0.01	-0.3935	<0.01	-0.5184	0.01	-0.8990*	0.01
	Future temperature changes (℃)	0.5791	0.18	0.3461	0.48	0.3203	0.59	0.4863	0.44
Intentional Injury	Proportion of population over 60 years (%)	0.1001	0.09	0.0990	0.10	0.1221	0.12	0.2232	0.11
	Percentage of illiterate over 15 years (%)	0.0148	0.43	0.0164	0.41	0.0276	0.31	0.0448	0.36
	Unemployment rate (%)	-0.1989	0.25	-0.1765	0.33	-0.2381	0.33	-0.4334	0.32
	Disposable income per capita (×10^4 Yuan)	-0.3744	<0.01	-0.4097	<0.01	-0.5519	<0.01	-0.9608	<0.01
	Future temperature changes (℃)	0.3916	0.30	0.1470	0.72	0.0668	0.89	0.1716	0.74

The meta-regression models were used to explore the impact of socio-economic factors on the projected rate of injury attributable to temperature change in the future. The statistical tests were two-sided with values of *P*<0.05 as statistical significance.

Table S7. The projected number of injury deaths attributable to temperature change in the 2060s and 2090s compared to the 2010s under different RCP scenarios 33 provinces in China

	RCP 4.5		RCP8.5	
	2060s	2090s	2060s	2090s
**Total Injury**				
Beijing	403(113,709)	444(124,783)	551(154,972)	944(264,1669)
Tianjin	514(156,894)	585(177,1021)	740(224,1292)	1254(379,2192)
Hebei	3776(954,6344)	4175(1053,7026)	5377(1355,9058)	9129(2299,15383)
Shanxi	1418(368,2456)	1562(405,2714)	2034(526,3540)	3487(901,6077)
Inner Mongolia	1072(226,1876)	1146(241,2009)	1499(315,2633)	2540(533,4465)
Liaoning	1444(407,2554)	1556(438,2756)	2029(570,3600)	3444(966,6118)
Jilin	784(205,1372)	853(222,1497)	1133(295,1996)	1930(501,3410)
Heilongjiang	1260(345,2300)	1378(377,2518)	1849(504,3385)	3172(865,5813)
Shanghai	537(110,934)	540(111,940)	757(155,1321)	1305(267,2278)
Jiangsu	3355(898,6009)	3352(896,6009)	4670(1247,8391)	8139(2172,14637)
Zhejiang	2282(664,4169)	2411(700,4410)	3377(978,6189)	5862(1696,10761)
Anhui	3193(818,5377)	3207(821,5404)	4413(1128,7452)	7798(1991,13185)
Fujian	1441(413,2501)	1724(493,2997)	2257(644,3928)	3886(1107,6771)
Jiangxi	2407(618,4098)	2711(695,4624)	3491(893,5963)	6139(1567,10506)
Shandong	4170(1044,7395)	4442(1111,7887)	6094(1522,10833)	10243(2558,18215)
Henan	4747(1324,8449)	4927(1373,8782)	6676(1857,11921)	11526(3202,20617)
Hubei	3989(1074,7055)	4194(1128,7426)	5426(1457,9622)	9525(2554,16914)
Hunan	3708(795,6608)	4202(899,7506)	5383(1149,9632)	9480(2018,17009)
Guangdong	2403(693,4066)	3034(873,5148)	4256(1222,7234)	7215(2069,12277)
Guangxi	1994(532,3510)	2468(656,4358)	3300(876,5837)	5554(1470,9849)
Hainan	225(61,389)	269(73,468)	391(106,679)	643(175,1116)
Chongqing	1097(265,1911)	1258(303,2197)	1625(391,2843)	2877(692,5040)
Sichuan	3583(1091,6284)	4212(1281,7395)	5848(1777,10276)	10176(3094,17875)
Guizhou	1644(430,2871)	1973(514,3457)	2568(668,4509)	4465(1158,7862)
Yunnan	2543(687,4578)	3139(847,5655)	4530(1222,8170)	7858(2122,14157)
Tibet	189(47,333)	217(54,382)	314(78,552)	529(132,928)
Shaanxi	1670(421,2918)	1766(445,3091)	2454(617,4303)	4197(1054,7370)
Gansu	1278(354,2216)	1379(382,2393)	1954(540,3396)	3309(915,5750)
Qinghai	330(91,570)	377(104,651)	542(150,939)	936(258,1618)
Ningxia	350(93,617)	377(100,666)	519(138,919)	885(234,1570)
Xinjiang	1296(285,2277)	1467(322,2581)	2064(452,3639)	3477(762,6129)
Hongkong	133(26,230)	166(33,286)	233(46,404)	397(79,688)

Macao	8(2,15)	10(3,18)	15(4,26)	25(7,44)
**Unintentional Injury**				
Beijing	346(56,638)	381(62,705)	472(77,875)	809(131,1502)
Tianjin	343(52,613)	391(59,699)	494(75,884)	837(126,1499)
Hebei	2527(30,4800)	2792(33,5314)	3595(43,6849)	6103(73,11631)
Shanxi	1212(96,2294)	1335(106,2533)	1738(137,3303)	2979(235,5668)
Inner Mongolia	775(89,1461)	828(95,1564)	1082(123,2048)	1834(209,3471)
Liaoning	1112(189,2016)	1197(203,2175)	1561(264,2840)	2648(447,4824)
Jilin	553(51,1031)	601(55,1123)	799(73,1496)	1359(124,2553)
Heilongjiang	983(99,1905)	1074(108,2085)	1441(145,2802)	2472(248,4808)
Shanghai	412(35,783)	414(36,787)	581(50,1106)	1001(86,1906)
Jiangsu	2356(256,4620)	2354(256,4617)	3278(355,6438)	5712(619,11227)
Zhejiang	1626(194,3130)	1718(205,3309)	2404(286,4638)	4172(495,8058)
Anhui	1982(298,3868)	1990(299,3887)	2737(410,5356)	4836(724,9474)
Fujian	1056(55,2047)	1263(66,2453)	1652(86,3215)	2843(148,5542)
Jiangxi	1499(221,2909)	1687(249,3281)	2172(319,4231)	3817(560,7450)
Shandong	2687(401,5111)	2862(427,5448)	3925(585,7480)	6596(983,12575)
Henan	2928(184,5457)	3038(191,5669)	4115(259,7690)	7101(446,13290)
Hubei	2247(154,4165)	2362(161,4381)	3055(208,5672)	5360(365,9963)
Hunan	2296(272,4377)	2600(308,4969)	3330(393,6373)	5860(691,11244)
Guangdong	1587(235,3041)	2002(295,3854)	2807(413,5417)	4757(699,9198)
Guangxi	1334(131,2461)	1650(162,3055)	2205(216,4092)	3709(362,6900)
Hainan	153(13,298)	184(15,358)	267(22,520)	438(37,854)
Chongqing	1000(102,1945)	1147(117,2235)	1481(151,2892)	2621(267,5124)
Sichuan	2797(213,5212)	3287(250,6133)	4562(346,8520)	7940(603,14824)
Guizhou	1209(106,2268)	1449(126,2730)	1885(164,3560)	3274(284,6205)
Yunnan	1630(192,3093)	2011(237,3819)	2901(341,5515)	5034(593,9560)
Tibet	145(10,272)	166(11,313)	240(16,452)	405(28,761)
Shaanxi	1234(181,2303)	1304(191,2437)	1811(264,3392)	3097(451,5809)
Gansu	764(88,1444)	825(95,1559)	1168(135,2211)	1978(228,3744)
Qinghai	257(21,492)	293(24,561)	422(35,809)	728(60,1395)
Ningxia	279(30,523)	301(32,564)	413(44,778)	704(76,1327)
Xinjiang	1053(38,1925)	1191(43,2182)	1675(61,3075)	2822(102,5179)
Hongkong	68(8,133)	84(10,166)	119(14,234)	202(24,399)
Macao	4(1,8)	5(1,10)	8(1,14)	13(1,25)
**Intentional Injury**				

Beijing	174(86,277)	193(94,308)	240(117,383)	412(201,659)
Tianjin	160(81,257)	183(93,295)	232(117,374)	394(199,635)
Hebei	1291(632,2004)	1432(700,2232)	1848(902,2886)	3140(1532,4907)
Shanxi	485(238,773)	537(263,858)	701(343,1123)	1204(588,1934)
Inner Mongolia	462(222,704)	495(237,756)	649(310,993)	1102(525,1684)
Liaoning	695(348,1046)	752(375,1137)	984(490,1493)	1675(832,2551)
Jilin	395(192,618)	431(209,678)	576(279,910)	985(475,1565)
Heilongjiang	612(292,952)	671(320,1046)	904(429,1413)	1553(737,2433)
Shanghai	178(87,277)	179(88,279)	252(123,393)	435(213,678)
Jiangsu	983(492,1493)	983(491,1495)	1374(685,2093)	2397(1194,3654)
Zhejiang	592(281,901)	627(297,957)	881(416,1351)	1535(723,2359)
Anhui	1503(726,2304)	1512(729,2320)	2087(1004,3213)	3697(1774,5705)
Fujian	467(230,716)	560(275,862)	736(361,1135)	1271(621,1964)
Jiangxi	608(268,922)	688(302,1047)	889(389,1356)	1569(684,2400)
Shandong	1933(926,2999)	2063(986,3210)	2837(1353,4423)	4771(2274,7445)
Henan	1790(908,2706)	1861(943,2817)	2529(1277,3840)	4378(2205,6659)
Hubei	2338(1142,3644)	2463(1202,3844)	3195(1557,5002)	5624(2735,8821)
Hunan	1108(574,1718)	1261(651,1961)	1620(834,2526)	2866(1469,4486)
Guangdong	819(391,1281)	1040(494,1634)	1464(693,2306)	2487(1176,3925)
Guangxi	519(239,844)	646(296,1057)	867(396,1422)	1467(666,2411)
Hainan	96(45,150)	116(54,180)	168(79,262)	276(129,431)
Chongqing	484(236,744)	557(271,860)	722(351,1117)	1280(621,1985)
Sichuan	1351(635,2098)	1592(747,2477)	2215(1038,3454)	3850(1805,6001)
Guizhou	654(327,1033)	790(392,1256)	1032(511,1648)	1803(889,2893)
Yunnan	1047(519,1598)	1295(640,1979)	1872(924,2865)	3241(1603,4952)
Tibet	56(21,93)	65(24,107)	94(34,155)	157(58,259)
Shaanxi	598(269,921)	634(284,977)	884(395,1368)	1515(676,2350)
Gansu	457(224,713)	494(242,771)	702(343,1100)	1189(580,1861)
Qinghai	129(63,205)	147(72,235)	212(104,340)	366(179,584)
Ningxia	101(44,161)	109(48,174)	151(66,242)	258(112,414)
Xinjiang	422(196,667)	479(222,758)	676(312,1075)	1138(525,1809)
Hongkong	109(52,176)	135(65,219)	191(92,310)	326(156,529)
Macao	7(3,11)	8(4,14)	12(6,20)	20(10,34)

Table S8. The sensitivity analysis of the excess risk(%) for each 1℃ increase in daily temperature on total injury death.

Sensitivity analysis	Variables changed	ER (95% CI)
Adjusting for different pollutants	$PM_{2.5}$	0.50(0.13, 0.88)
	PM_{10}	0.49(0.12, 0.87)
	SO_2	0.50(0.12, 0.88)
	NO_2	0.52(0.13, 0.90)
	CO	0.50(0.13, 0.87)
Changing max lag days of temperature (day)	0	0.52(0.14, 0.90)
	1	0.50(0.13, 0.88)
	2	0.45(0.08, 0.82)
Using moving average of daily mean temperature	lag01	0.48(0.11, 0.86)
Changing the indicator of daily temperature	Minimum temperature	0.53(0.35, 0.72)
	Maximum temperature	0.52(0.39, 0.66)

Table S9. The sensitivity analysis of the projected number of injury deaths attributable to temperature change in the 2060s and 2090s compared to the 2010s under different RCP scenarios

Sensitivity analysis	Item	RCP 4.5		RCP8.5	
		2060s	2090s	2060s	2090s
Shared Socioeconomic Pathways in future population	SSP1	48016(13325,86535)	35732(9911,64428)	71484(19829,128898)	82423(22904,148337)
	SSP2	50182(13926,90439)	40512(11237,73049)	74724(20727,134740)	93445(25967,168172)
	SSP3	53501(14847,96421)	30932(8580,55774)	79704(22109,143719)	71353(19828,128414)
	SSP4	45098(12515,81277)	30932(8580,55774)	67115(18617,121020)	71353(19828,128414)
	SSP5	48031(13329,86562)	35760(9919,64480)	71507(19835,128939)	82490(22923,148456)
General Circulation Models in future temperature	GFDL-ESM2M	36729(8858,63680)	33516(8098,57998)	50774(12217,88238)	93972(22549,163746)
	HadGEM2-ES	74815(17934,130488)	82444(19757,143832)	112394(26976,195809)	165442(39776,287772)
	IPSL-CM5A-LR	65998(15844,114952)	71431(17137,124488)	103024(24731,179458)	179695(43229,312389)
	MIROC-ESM-CHEM	71179(17081,124020)	85947(20615,149821)	107218(25715,186913)	201741(48591,350316)
	NorESM1-M	58020(13937,100998)	66135(15863,115285)	83988(20140,146436)	142078(34125,247355)
Average of 5 GCMs and population in 2017		61348(14731,106828)	67895(16294,118285)	91480(21956,159371)	156586(37654,272316)

Table S10. The sensitivity analysis of the projected rate (per 100 000) of injury deaths attributable to temperature change in the 2060s and 2090s compared to the 2010s under different RCP scenarios

Sensitivity analysis	Item	RCP 4.5		RCP8.5	
		2060s	2090s	2060s	2090s
Shared Socioeconomic Pathways in future population	SSP1	4.41(1.22,7.95)	4.88(1.35,8.8)	6.57(1.82,11.85)	11.25(3.13,20.25)
	SSP2	4.41(1.22,7.95)	4.88(1.35,8.8)	6.57(1.82,11.85)	11.26(3.13,20.26)
	SSP3	4.41(1.22,7.95)	4.88(1.35,8.8)	6.57(1.82,11.85)	11.25(3.13,20.25)
	SSP4	4.41(1.22,7.95)	4.88(1.35,8.8)	6.57(1.82,11.84)	11.25(3.13,20.25)
	SSP5	4.41(1.22,7.95)	4.88(1.35,8.8)	6.57(1.82,11.85)	11.25(3.13,20.25)
General Circulation Models in future temperature	GFDL-ESM2M	3.30(2.31,4.25)	3.01(2.11,3.87)	4.57(3.19,5.89)	8.46(5.90,10.93)
	HadGEM2-ES	6.74(4.70,8.71)	7.43(5.17,9.60)	10.12(7.06,13.07)	14.88(10.39,19.21)
	IPSL-CM5A-LR	5.94(4.14,7.67)	6.43(4.48,8.31)	9.27(6.47,11.98)	16.16(11.29,20.85)
	MIROC-ESM-CHEM	6.41(4.47,8.28)	7.74(5.40,10.00)	9.65(6.73,12.47)	18.14(12.67,23.39)
	NorESM1-M	5.22(3.64,6.74)	5.95(4.15,7.69)	7.56(5.27,9.77)	12.79(8.92,16.51)
Average of 5 GCMs and population in 2017		4.41(1.06,7.69)	4.88(1.17,8.51)	6.58(1.58,11.46)	11.26(2.71,19.59)

Table S11. The assumption of five Shared Socioeconomic Pathways and the variables for future population projection in China

SSP	Assumption in future	Variables				Populations (million)	
		**Fertility**	**Mortality**	**Migration**	**Education**	**2060s**	**2090s**
SSP1	Moving toward a more sustainable path	Low	Low	Medium	High (FT-GET)	1088.04	732.42
SSP2	The middle of the road scenario	Medium	Medium	Medium	Medium (GET)	1137.12	830.09
SSP3	A fragmented world with an emphasis on security at the expense of international development	High	High	Low	Low (CER)	1212.32	1043.58
SSP4	A world of high inequalities, both between and within countries	Low	Medium	Medium	CER-10%/GET	1021.94	634.11
SSP5	A world that stresses technological progress and where economic growth is fostered by rapid development of human capital	Low	Low	High	High (FT-GET)	1088.39	733.01

Note: GET refers the Global Education Trend scenario; CER: the Constant Enrollment Rates; FT-GET: the arithmetic mean of the education progression rates implied under the Global Education Trend and Fast Track scenarios.

Table S12. The classifications of injured death by intention and mechanism

Cause	Intention	Mechanism
Total injury (V00-Y98)	Unintentional injury (V00-X59)	Transport injury (V00-V99)
		Fall (W00-W19)
		Mechanical force (W20-W64)
		Drowning (W65-W74)
		Suffocation (W75-W84)
		Poisoning (X40-X49)
		Other unintentional injury (W85-W99, X50-X59)
	Intentional injury (X60-Y09)	Suicide (X60-X84)
		Assault (X85-Y09)
	Other injury (Y10-Y98)	

Table S13. R-square and root-mean-square error of each pollutant

Pollutant	R-square	root-mean-square error
PM_{10}	0.89	13.74 μg/m^3
$PM_{2.5}$	0.91	8.89 μg/m^3
NO_2	0.80	8.22 μg/m^3
SO_2	0.81	8.76 μg/m^3
CO	0.63	0.25 mg/m^3
