# Supplementary material for: Temperature-related mortality in China from specific injury
Source: Nat Commun. 2023 Jan 3;14:37. doi: 10.1038/s41467-022-35462-4 (PMC9810693; doi:10.1038/s41467-022-35462-4)
Supplement: Supplementary file 2 — Reporting Summary [file 41467_2022_35462_MOESM2_ESM.pdf]

nature portfolio

Corresponding author(s): Wenjun Ma

Last updated by author(s): Jun 11, 2022

Reporting Summary

Nature Portfolio wishes to improve the reproducibility of the work that we publish. This form provides structure for consistency and transparency in reporting. For further information on Nature Portfolio policies, see our Editorial Policies and the Editorial Policy Checklist.

Statistics

For all statistical analyses, confirm that the following items are present in the figure legend, table legend, main text, or Methods section.

n/a	Confirmed	
☐	☒	The exact sample size (*n*) for each experimental group/condition, given as a discrete number and unit of measurement
☒	☐	A statement on whether measurements were taken from distinct samples or whether the same sample was measured repeatedly
☐	☒	The statistical test(s) used AND whether they are one- or two-sided<br>*Only common tests should be described solely by name; describe more complex techniques in the Methods section.*
☐	☒	A description of all covariates tested
☐	☒	A description of any assumptions or corrections, such as tests of normality and adjustment for multiple comparisons
☐	☒	A full description of the statistical parameters including central tendency (e.g. means) or other basic estimates (e.g. regression coefficient) AND variation (e.g. standard deviation) or associated estimates of uncertainty (e.g. confidence intervals)
☐	☒	For null hypothesis testing, the test statistic (e.g. *F*, *t*, *r*) with confidence intervals, effect sizes, degrees of freedom and *P* value noted<br>*Give P values as exact values whenever suitable.*
☒	☐	For Bayesian analysis, information on the choice of priors and Markov chain Monte Carlo settings
☒	☐	For hierarchical and complex designs, identification of the appropriate level for tests and full reporting of outcomes
☒	☐	Estimates of effect sizes (e.g. Cohen's *d*, Pearson's *r*), indicating how they were calculated

Our web collection on statistics for biologists contains articles on many of the points above.

Software and code

Policy information about availability of computer code

Data collection	All data were prepared using R project software (version 4.0.2).
Data analysis	All data were analysed using R software, with the "dlnm"(2.4.7) and "mvmeta" (1.0.3) packages used for model construction and the "ggplot2" (3.3.6) package used for figure plotting. The code that supports the findings of this study is available upon request (send requests to mawj@gdiph.org.cn).

For manuscripts utilizing custom algorithms or software that are central to the research but not yet described in published literature, software must be made available to editors and reviewers. We strongly encourage code deposition in a community repository (e.g. GitHub). See the Nature Portfolio guidelines for submitting code & software for further information.

Data

Policy information about availability of data

All manuscripts must include a data availability statement. This statement should provide the following information, where applicable:

- Accession codes, unique identifiers, or web links for publicly available datasets
- A description of any restrictions on data availability
- For clinical datasets or third party data, please ensure that the statement adheres to our policy

The original datasets generated or analyzed, or both, during this study are not publicly available because of governance restrictions and the identifiable nature of the data. Requests for access to raw data should be addressed to the corresponding author (mawj@gdiph.org.cn) and will be answered within 12 weeks. Meteorological data for the monitoring stations are available at the China Meteorological Data Sharing Service System (http://data.cma.cn/). Projected temperature

of CMIP5 GCM data are available Inter-Sectoral Impact Model Intercomparison Project (https://www.isimip.org). Air pollution monitoring data is available National Urban Air Quality Real-time Publishing Platform (https://air.cnemc.cn:18007/). The future populations under five SSPs are available from International Institute for Applied Systems Analysis (https://iiasa.ac.at). Data on province characteristics are available on the government's statistic yearbooks(http://www.stats.gov.cn/tjsj/ndsj/). The population density data are available from GeoData Institute in University of Southampton (http://www.worldpop.org.uk), and the GIS covariates are available from the Data Center for Resources and Environmental Sciences (http://www.resdc.cn/). The summary statistics and descriptive tables in this study are provided in the Supplementary Information.

Human research participants

Policy information about studies involving human research participants and Sex and Gender in Research.

Reporting on sex and gender	This study collected all injury death records from Disease Surveillance Points System (DSPS) in Guangdong (2013-2018), Hunan (2013-2018), Zhejiang (2013-2018), Yunnan (2013-2018), Tibet (2013-2019), and Jilin (2013-2018). Individual information including date of death, cause of death, residential address (sub-district level), gender, and age for each case was recorded.
Population characteristics	This study collected all injury death records from Disease Surveillance Points System (DSPS) in Guangdong (2013-2018), Hunan (2013-2018), Zhejiang (2013-2018), Yunnan (2013-2018), Tibet (2013-2019), and Jilin (2013-2018). During the study period, there are 609,827 injury deaths in six provinces of China with 504,040(82.65%) unintentional cases and 75,893 (12.45%) intentional cases. The number of injury deaths was much higher for males (403,701, 66.20%) than for females (206,104, 33.79%), and population aged over 50 years accounts for 59.50% of total injury deaths.
Recruitment	Not applicable.
Ethics oversight	This study was approved by the Ethics Committee of Guangdong Provincial Center for Disease Control and Prevention (No. 2019025).

Note that full information on the approval of the study protocol must also be provided in the manuscript.

Field-specific reporting

Please select the one below that is the best fit for your research. If you are not sure, read the appropriate sections before making your selection.

☐ Life sciences ☐ Behavioural & social sciences ☒ Ecological, evolutionary & environmental sciences

For a reference copy of the document with all sections, see nature.com/documents/nr-reporting-summary-flat.pdf

Ecological, evolutionary & environmental sciences study design

All studies must disclose on these points even when the disclosure is negative.

Study description	In the present study, we used over 600,000 injury deaths from six provinces in China to assess the association between temperature and injury mortality, and further to project the temperature-related injury mortality burden driven by climate change in the future.
Research sample	Combining the regional distribution (central, southern, northern, eastern and western China) and data accessibility in China, we collected all injury death records from the Disease Surveillance Points System in Guangdong, Hunan, Zhejiang, Yunnan, Tibet and Jilin. These death records represent the entire population of these six provinces. During the study period, there are 609,827 injury deaths in six provinces of China. Of these, 504,040 were unintentional injuries and 75,893 were intentional; 403,701 were male and 206,104 were female; 17,905 were 0-4 years, 16,591 were 5-14 years, 212,483 were 15-49 years, 16,7514 were 50-69 years and 195,334 were over 70 years.
Sampling strategy	All injury deaths recorded in the six provinces were included in this study.
Data collection	Injury death records were collected from Disease Surveillance Points System (DSPS) in Guangdong Province, Hunan Province, Zhejiang Province, Yunnan Province, Tibet Autonomous Region, and Jilin Province. All causes of death are diagnosed by physicians, with coded by the International Classification of Diseases tenth Revision (ICD-10), and the information of the deceased was reported to the DSPS and quality-controlled by professionals. Meteorological data in 698 monitoring stations across China was collected from the China Meteorological Data Sharing Service System (http://data.cma.cn/). Projected daily temperatures across China were obtained from the dataset of Coupled Model Intercomparison Project phase 5 (CMIP5), which is developed by Inter-Sectoral Impact Model Intercomparison Project (ISI-MIP, https://www.isimip.org). The monitoring station data of daily mass concentration of ambient air pollutants were collected from the National Urban Air Quality Real-time Publishing Platform (https://air.cnemc.cn:18007/). Data on population, employment, education, and income of each province in 2017 were collected from the National Statistical Yearbook. The population density data in 2015 were obtained from GeoData Institute in University of Southampton (http://www.worldpop.org.uk), and the geographic information system (GIS) covariates (geographic map, road density, land use data and GDP per capita) were obtained from the Data Center for Resources and Environmental Sciences (http://www.resdc.cn/). The future populations under five Shared Socioeconomic Pathways (SSPs, SSP1-SSP5) were collected from International Institute for Applied Systems Analysis (https://iiasa.ac.at).
Timing and spatial scale	Due to data accessibility, all individual injury death records through the DSPS in six provinces in China were collected, including

	Guangdong (2013-2018), Hunan (2013-2018), Zhejiang (2013-2018), Yunnan (2013-2018), Tibet (2013-2019), and Jilin (2013-2018). To match the injury mortality data in the spatial and temporal dimensions, daily meteorological monitoring data were collected during the study period and interpolated to a spatial resolution of 1km × 1km. Daily air pollution monitoring data were collected during the study period and land-use regression were used to obtain the individual levels; projected daily temperatures for 2010-2100 were bias-corrected and downscaled at a 0.5° × 0.5° spatial resolution.
Data exclusions	No data were excluded from the analyses.
Reproducibility	The study was based on data collected for model building to assess the effect of temperature on injury death, and to projected the temperature-related injury mortality burden in the future. Where the model and parameters were determined, the results were unequivocal for all replicate experiments. In addition, we also performed 19 sensitivity analyzes with similar results.
Randomization	This study is a case-crossover design based on all injury deaths recorded in six provinces. For each injury death, exposure to daily mean temperature on the day of death occurred (case day) was compared to the exposure on the same days of the week in the same calendar month (control days). This method of matching case and controls can effectively control a series of confounding, such as long-term and seasonal trends, influence of day-of-week, sex, age, economic conditions, and lifestyle. In addition, we controlled for covariates such as relative humidity, air pollution and holiday in the model.
Blinding	This study is a case cross-over design. Blinding was not applicable to the self-control data in this study.

Did the study involve field work? ☐ Yes ☒ No

Reporting for specific materials, systems and methods

We require information from authors about some types of materials, experimental systems and methods used in many studies. Here, indicate whether each material, system or method listed is relevant to your study. If you are not sure if a list item applies to your research, read the appropriate section before selecting a response.

Materials & experimental systems

n/a	Involved in the study
☒	☐ Antibodies
☒	☐ Eukaryotic cell lines
☒	☐ Palaeontology and archaeology
☒	☐ Animals and other organisms
☒	☐ Clinical data
☒	☐ Dual use research of concern

Methods

n/a	Involved in the study
☒	☐ ChIP-seq
☒	☐ Flow cytometry
☒	☐ MRI-based neuroimaging
